# 3D-Printed Microneedle Patch for the Treatment of Melanoma via Synergistic Chemotherapy and Photothermal Therapy

**DOI:** 10.1021/acsabm.5c01606

**Published:** 2026-01-26

**Authors:** Hilal Yilmaz, Louna Karzoun, Berfin Ilayda Ozturk Guzelcan, Hakan Sahin, Yagmur Kazancioglu, Mohammad Yaman Habra, Esra Yuca Yilmaz, Elif Guzel, Oguzhan Gunduz, Yavuz Nuri Ertas, Cem Bulent Ustundag

**Affiliations:** † Department of Bioengineering, Faculty of Chemical and Metallurgical Engineering, 52999 Yildiz Technical University, Istanbul 34210, Türkiye; ‡ Health Biotechnology Joint Research and Application Center of Excellence (SABIOTEK), Esenler, Istanbul 34220, Turkey; § Center for Nanotechnology & Biomaterials Application and Research (NBUAM), 52982 Marmara University, Istanbul 34722, Türkiye; ∥ Department of Molecular Biology and Genetics, Faculty of Science and Literature Department, Yildiz Technical University, Istanbul 34210, Turkey; ⊥ Department of Biomedical Engineering, 52958 Erciyes University, Kayseri 38039, Türkiye; # ERNAM−Nanotechnology Research and Application Center, Erciyes University, Kayseri 38039, Türkiye; ¶ Department of Histology and Embryology, Cerrahpasa Faculty of Medicine, 532719 Istanbul University-Cerrahpasa, Istanbul 34098, Türkiye; ∇ Department of Biomedical Engineering, Faculty of Electrical and Electronics, Yildiz Technical University, Istanbul 34220, Türkiye; ○ Department of Metallurgical and Materials Engineering, Faculty of Technology, Marmara University, Istanbul 34722, Türkiye

**Keywords:** melanoma, microneedle, 3D-printing, 5-fluorouracil, photothermal therapy

## Abstract

Melanoma is a malignant type of skin cancer that originates from pigment-producing cells called melanocytes. Alongside its aggressive trajectory, it is characterized by metastasis. The lack of targeting ability and high toxicity in traditional chemotherapy, along with issues such as the dermal barrier and patient compliance, necessitate local and synergistic treatment approaches. Patches that are part of transdermal drug delivery systems and use hydrogel microneedles to deliver drugs noninvasively, locally, and synergistically, are a recently emerging treatment alternative. In this study, we designed a microneedle patch composed of microneedles produced by 3D digital light processing, which were made of sodium alginate and GelMA. The GelMA support base contained an anticancer drug (5-FU) and graphene oxide quantum dots dispersed in a polyvinylpyrrolidone matrix. Quantum dots conferred photothermal activity under near-infrared (808 nm) light, whereas 5-FU provided the chemotherapy effect. The microneedle had a height of 917.6 ± 47 μm, tip radius of 26.9 ± 0.4 μm, 5-FU burst release of 63 ± 0.665% within the first hour, and 100% release within 96 h. It exhibited photothermal properties, reaching 46.3 °C within 5 min under the effect of NIR. The patch substantially reduced the viability of cancerous A375 cells, exhibiting suitable mechanical properties for skin penetration, as well as swelling and degradation properties for drug release. The findings suggest that the minimally invasive microneedle platform, which enhances patient compliance, could be a promising solution for melanoma treatment through the synergistic use of chemotherapy and photothermal therapy.

## Graphical abstract

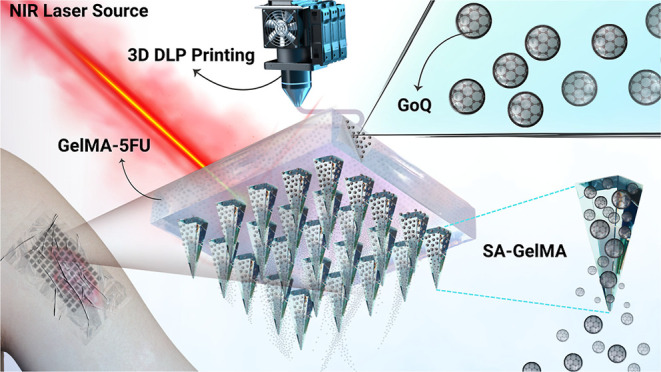

## 1 Introduction

Skin cancer is the most common type of cancer worldwide. It can result from a combination of genetic and environmental factors, with long-term exposure to ultraviolet (UV) light being the most common cause.^1^ It is a major global health issue that has significant psychosocial effects and requires substantial investment in treatments and technologies.^2^ Melanoma, a type of skin cancer, is among the most aggressive cancers and is susceptible to metastasis, complicating current clinical treatment.^3^ It is the sixth most often diagnosed cancer and accounts for a concerning 80% skin cancer-related fatalities. The primary treatment for early stage melanoma involves surgical removal of the tumor. Surgical excision is effective for early localized tumors, but it is not suitable for metastatic melanoma, frequently resulting in incomplete tumor removal and potential damage to surrounding healthy tissues. For intermediate and advanced stages of melanoma, other treatments such as chemotherapy, radiation therapy, immunotherapy, and targeted therapy are often required, yet chemotherapy becomes the primary treatment at this stage.^4^ However, the parenteral administration of chemotherapy may induce anxiety, discomfort, and heightened infection risks, particularly in immunocompromised cancer patients. Traditional chemotherapy, though occasionally beneficial, suffers from systemic toxicity, low response rates, and the emergence of drug resistance, which significantly limits its clinical utility.^5^ Notwithstanding advancements in cancer research and treatment strategies, the management of melanoma continues to provide a considerable challenge. This is mainly attributable to its aggressive characteristics, potential for metastasis, and intrinsic resistance to conventional chemotherapeutic agents.

While the search for ideal treatments continues, transdermal drug delivery systems (TDDS) appear to be the most promising method for the localized, self-administered delivery of drugs, which can enhance drug bioavailability in the treatment of melanoma. This method can deliver bioactive agents dermally or subcutaneously in a noninvasive manner.^6^ Thus, the bioavailability of bioactive agents escaping the gastrointestinal tract may increase. Microneedles can penetrate the epidermis while avoiding contact with capillaries and nerves, making them one of the most popular TDDS.^7^ The microchannels opened by microneedles facilitate the diffusion of bioactive agents, providing stable and continuous therapeutic effects. In cancer treatment, microneedles can easily provide localized distribution of bioactive agents, increasing drug concentrations in tumor regions while reducing toxicity to healthy tissues. Not only do microneedles offer a convenient method for delivering drugs effectively and relatively painlessly, but they can also be combined with multimodal strategies, such as photothermal therapy, immunotherapy, and gene therapy, to achieve synergistic efficacy.^8–11^

Recently, 3D printing has attracted growing interest for fabricating hydrogel microneedles due to its ability to precisely tailor microneedle array topology and geometry.^12–14^ 3D printing technology enables the complete customization of microneedle geometry, including array properties such as size and density, as well as individual properties like shape and length. Furthermore, 3D printing can produce faster and more complex geometries than traditional manufacturing methods.^15^ Hydrogel microneedles produced using a 3D digital light processing (DLP) printing system can perform multifunctional tasks, such as maximizing drug release with a minimum footprint, by taking advantage of their sharp protrusion and microporous structure.^16^ DLP is a self-assembled, high-precision 3D printing system based on light curing, has high printing accuracy, and can create precise microneedles.^17,18^ This system helps design microneedle platforms specific to the damaged area. It allows 3D models of microneedles to be conveniently and economically modified and sliced. The DLP system is inexpensive, which significantly reduces production costs compared to other methods.^19^

Hydrogels produced from the natural polysaccharide sodium alginate (SA) are among the most widely studied natural polymers due to their properties, including low toxicity, biocompatibility, and nonimmunogenicity.^20^ They can also be physically cross-linked in the presence of divalent cations, such as calcium.^21^ SA is also a cheap, biocompatible biomaterial that cross-links rapidly and gently and is widely used in soft tissue repair and regeneration.^22^ However, although SA is suitable for use as a patch, a different natural polymer with valuable properties is also needed to overcome the limitations of using a single polymer formulation. Hydrogels with reinforced composite structures enable the development and optimization of hydrogel properties, thereby expanding their areas of application. Gelatin methacryloyl (GelMA) is obtained by treating the amine group of gelatin, a natural, cell-compatible protein, with methacrylate anhydride.^23^ Gelatin is a special polymer with a bioactive Arg-Gly-Asp (RGD) chain that enables cell-active molecules to bind to the polymeric network. Cross-linking requires the addition of a photoinitiator that decomposes into radicals in the presence of UV light.^24^ Gelatin modification aims to obtain an irreversible, stable hydrogel in response to temperature changes.^25^

5-Fluorouracil (5-FU) is a Food and Drug Administration (FDA)-approved anticancer drug used for topical skin cancer treatment. Its mechanism of action is based on inhibiting the enzyme thymidylate synthetase, thereby interfering with DNA replication. Although 5-FU exhibits a response rate of roughly 10–15% in initial cancer therapies, its cytotoxic effects are enhanced following intracellular activation.^26^ Besides, 5-FU has a short half-life, which may require high injection doses and multiple applications. Specifically, high-dose local application can cause serious side effects, such as vasculitis, hyperpigmentation, erythema, purpura, burning sensation, and invasive injection causes intense pain.^27^ Although it is effective in treating cancers, its systemic use is restricted due to significant adverse effects. Topical administration of 5-FU has been suggested as an alternative method for treating skin cancer. Nevertheless, the inadequate permeability of 5-FU through the dermis remains a challenge, compounded by its propensity to provoke acute localized inflammation, thereby impacting patient adherence.^28^

Thanks to its excellent physicochemical properties, graphene oxide quantum dots (GoQD) are used in many applications, such as antimicrobial activity, bioimaging, phototherapy, drug delivery, tissue engineering, and biosensors.^29–32^ Photothermal therapy utilizes highly absorbing materials, which uptake energy from an irradiation source and dissipate it in the form of heat to trigger cell death.^33,34^ GoQD have high photothermal conversion efficiency, which can efficiently convert light energy into heat, leading to localized hyperthermia in tumors. Thus, GoQD were selected to functionalize the support base of the patch. However, this unique material must be conjugated with a polymeric matrix to ensure the particles are evenly distributed when coating the hydrogel. Therefore, polyvinylpyrrolidone (PVP) was preferred as the polymeric matrix material. This is because PVP is typically used as a stabilizing polymer in conjunction with graphene-based materials to improve distribution and enhance mechanical properties. Electrospraying is based on applying an electric field to a polymer solution, which is ejected from a syringe, yielding particles from the nano to micron scale, and it can be used to encapsulate GoQD with PVP, which results in a sustained and controlled release profile with improved encapsulation efficiency.^35,36^

In this study, a patch composed of 5-FU-containing GelMA microneedles and a SA-GelMA support, which contains PVP-encapsulated GoQD, was developed using the 3D DLP printing method. The microneedle patch enables easy penetration into the skin’s stratum corneum, facilitating the local release of 5-FU. In contrast, the support layer contributes to the enhanced mechanical stability of the patch and the controlled release of the photothermal agent GoQD. The synergistic effects of chemotherapy and photothermal therapy have been demonstrated as a practical approach for treating melanoma.

## 2 Materials and Methods

### 2.1 Materials

The following items were purchased from Sigma-Aldrich (Germany): polyvinylpyrrolidone (PVP) with a molecular weight of 40,000 Da; methacrylic anhydride (MAA); lithium phenyl-2,4,6-trimethylbenzoylphosphinate (LAP); 5-fluorouracil (5-FU); a dialysis membrane with a cutoff value of 14 kDa and an average flat width of 43 mm; and paraformaldehyde. Gelatin Type B (gel strength 230–250 g Bloom) from bovine skin was purchased from Halavet (Türkiye). Sodium carbonate, sodium hydroxide, 37% hydrochloric acid, and Parafilm were purchased from Merck (Germany). Sodium hydrogen carbonate (>99.7%), sodium alginate, and calcium chloride dihydrate were purchased from Isolab (Germany). Phosphate-buffered saline (PBS, pH 7.4) was purchased from ChemBio (Türkiye). l-glutamine (GlutaMAX), penicillin, streptomycin, and Fungizone (PSF), as well as fetal bovine serum (FBS), were supplied by Thermo Fisher Scientific (USA). DMEM/F-12 was purchased from Sartorius AG (Germany).

### 2.2 Methods

#### 2.2.1 Synthesis of GelMA

Commercially obtained bovine type B gelatin from bovine skin (10%, w/v) was dissolved in a 0.1 M bicarbonate buffer solution at 60 °C. Then, 0.2 mL of MAA per gram of gelatin was added to the solution, and the reaction was carried out at 50 °C for 3 h with continuous stirring. During this time, one-sixth of the MAA (334 μL) was added dropwise to the gelatin solution every 30 min, with vigorous stirring, and the pH was adjusted to 9–9.5 after each addition. This caused the free amino groups of the lysine and hydroxylysine amino acids in the gelatin to react with the MAA. To remove the unreacted MAA and methacrylic acid byproducts, the solution was distilled at 40 °C for 5 days and dialyzed in water using a 14 kDa molecular weight cutoff (MWCO) membrane. The dialysate was frozen at −20 °C for 24 h and then lyophilized for 4 days and stored at −20 °C until further use.^37,38^

#### 2.2.2 GelMA Degree of Substitution

In our previous study, the degree of substitution (DS) of GelMA was determined using two complementary techniques: proton nuclear magnetic resonance (^1^H NMR) spectroscopy (Bruker Avance Neo 500 MHz, Bremen, Germany) and the trinitrobenzenesulfonic acid (TNBS) assay.^39^ For ^1^H NMR analysis, gelatin and GelMA were dissolved in deuterium oxide (D_2_O) at a concentration of 10 mg/mL and analyzed at room temperature using a 500 MHz spectrometer.

For the TNBS assay, gelatin and GelMA samples were dissolved in 0.1 M sodium bicarbonate buffer (pH 5.8) to obtain a final concentration of 0.5 mg/mL. Subsequently, 250 μL of 0.01% (w/v) TNBS solution was added to 500 μL of each sample. The reaction mixtures were incubated at 40 °C for 2 h, after which 250 μL of 10% (w/v) sodium dodecyl sulfate (SDS) and 125 μL of 1 M hydrochloric acid were added to terminate the reaction. The concentration of free amino groups was quantified by measuring the UV absorbance at 335 nm using a UV–vis spectrophotometer (UV-1280, Shimadzu, Japan), with glycine standard solutions at concentrations of 0, 1, 5, and 10 μg/mL.

The DS value of GelMA was calculated according to the following eq 1.

1 $$DS\;\left(\%\right)=1-\frac{\left(peak\;area\;of\;GelMAlysine\;methtlene\right)}{\left(peak\;area\;of\;gelatinlysine\;methtlene\right)}\times 100$$

#### 2.2.3 Synthesis of SA-GelMA

For 1 mL of PBS, 0.2 g of GelMA was weighed and left to dissolve at 100 rpm and 50 °C for 10 min. Once the GelMA had completely dissolved, different concentrations of SA (0.5%, 1%, and 2%) were weighed and added to three separate GelMA solutions, which were then left to dissolve for 15 min. Later, three solutions were prepared for use in the 3D DLP printer: 0.5% SA-GelMA, 1% SA-GelMA, and 2% SA-GelMA. These solutions form the support base of the microneedle patches. For DLP printing, 2.5 mg of LAP per mL was mixed into the solutions at 50 °C until fully dissolved. Afterward, the solutions were transferred to the 3D DLP printer tank.

#### 2.2.4 Preparation of GelMA-5FU Solution

For 1 mL of PBS, 0.2 g of GelMA was weighed and dissolved at 100 rpm and 50 °C for 10 min 2.5 mg of LAP was added to the solution for DLP printing and allowed to dissolve completely. Once the heating process had stopped, the solution was quickly cooled, and 20 mg of 5-FU was added, ensuring it was thoroughly dissolved.

#### 2.2.5 Design of the Microneedle Patch using 3D DLP Printing

Printing was carried out using a DLP 3D printer (Anycubic Photon D2, Anycubic, China) with a fixed slicing profile to ensure reproducibility. Microneedle patches were fabricated from SA-GelMA (support base) and GelMA-5FU (drug-loaded microneedles). The patch geometry was designed using computer-aided design software (SolidWorks, France) as a 10 × 10 mm model with a total thickness of 2 mm, comprising a 1 mm supporting base and 1 mm conical microneedles (≈0.6 mm diameter, 1 mm height). The 1 mm needle height was selected to enable penetration through the stratum corneum and epidermis, reaching the upper dermis, which is the optimal region for transdermal drug diffusion and local therapeutic action. Hydrogel microneedles with a height of approximately 1 mm can successfully cross the skin barrier and deliver drugs directly into the dermal tissue without causing pain or reaching deep blood vessels.^40^ The additional 1 mm base provided mechanical support and structural stability during DLP printing, handling, and application. Therefore, the selected 2 mm total patch thickness ensures both efficient dermal delivery and adequate mechanical integrity during handling and skin insertion.

The design was exported as an STL file and sliced in Chitubox (Shenzhen Chuangbide Technology Co., China). Printing was performed at 405 nm with a light intensity of 12 mW/cm^2^. The layer thickness was set to 0.020 mm (20 μm), with a normal exposure time of 70 s and an off time of 1 s per layer. To enhance initial adhesion, a bottom exposure time of 120 s for 6 bottom layers was applied. Separation/recoating kinematics were kept constant using a Z-lift distance of 5.0 mm at a Z-lift speed of 2.0 mm/s and Z-retract speed of 3.0 mm/s. Antialiasing was enabled (level = 1). The printer’s optical engine provides a 2560 × 1440 projected pixel array and a native *XY* pixel size of ∼51 μm, which defines the in-plane printing resolution for lateral feature fidelity.

To create the bilayer structure, the SA–GelMA base solution (at varying concentrations) was first poured into the printing tank, and printing was initiated. When the print reached ∼50% completion, the process was stopped; the vat was changed with a clean one, and the GelMA–5FU solution was then poured into the tank to fabricate the microneedles, and printing was resumed to completion, yielding a double-layer patch (support base and microneedle array) (Figure 1). For ionic cross-linking and physical bonding of the prepared patches containing SA, a 0.1 M calcium chloride (CaCl_2_) solution was prepared by dissolving 0.056 g of CaCl_2_ in 4 mL of pure water. The volume was then completed to 5 mL by adding pure water and stirring until the solution was fully dissolved. Ionic cross-linking was achieved by immersing the patches in the prepared 0.1 M CaCl_2_ solution for 15 min.^41^ Finally, a washing step with PBS was performed to remove excess calcium ions from the patches.

**Figure 1:**
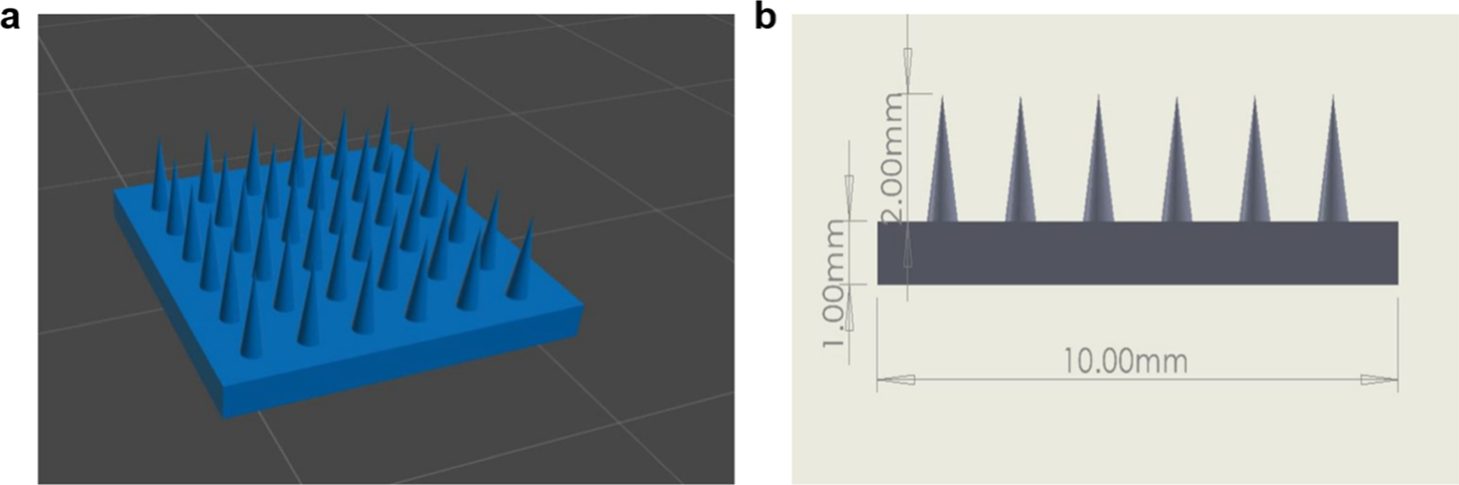
Design details: (a) chitubox image, (b) dimensions of the microneedle patch.

#### 2.2.6 Synthesis of GoQD and Preparation of PVP-GoQD

GoQD synthesis was carried out in four stages: reaction, reaction quenching, washing, and drying. 360 mL of sulfuric acid (H_2_SO_4_) and 40 mL of phosphoric acid (H_3_PO_4_) were placed in a beaker, which was then placed in an oil bath. The oil temperature was adjusted to between 55 and 60 °C, while the oil level was above the liquid mixture. This ensured that the reaction temperature was fixed at 40–45 °C. The beaker was then placed in a mixer in the oil bath and mixed at 200 rpm. 3 g of graphite were slowly added to the acid solution. Then, 18 g of potassium permanganate (KMnO_4_) was slowly added to the solution, and the temperature of the solution was measured frequently with a thermometer. The reaction was stirred at 40–45 °C for 16 h. Then, the reaction quenching process was carried out. The suspension, which had been mixed for 16 h by placing 400 g of ice in a large beaker, was transferred to an ice-filled beaker and mixed, ensuring that no graphene oxide remained in the original beaker. While the suspension was mixed with ice, 3 mL of 30% H_2_O_2_ was added dropwise. Next, the washing step was started. The mixture was centrifuged at 3000 rpm for 45 min. The centrifuge tubes were filled with 400 mL of pure water to wash the pellets and centrifuged at 5000 rpm for 45 min. Centrifugation was performed thrice with HCl and thrice with pure ethanol at 5000 rpm for 45 min. Finally, the drying step was performed. The nanoparticles were obtained by drying the Go solution in an oven. To produce GoQD, 10 mL of distilled water and 2 mL of ammonia are added to 5 mL of the Go solution in a protected, heat-resistant container, which was then mixed.^42^ After preparing GoQD from graphene oxide through 5 h of sonication, the obtained dispersion was centrifuged, and the supernatant was collected. Then, 50 μL of the supernatant was mixed with 1450 μL of distilled water and filtered through a 0.22 μm membrane before measurement.

GoQD were blended with PVP to coat the base of the microneedle patch using the electrospraying method. A 10% (w/v) PVP solution was prepared by slowly adding 10 mL of pure ethanol to a 100 mL solution of PVP at 200 rpm without heating. GoQD were then added at different concentrations (500 μL and 1250 μL), after which the mixture was homogenized by pipetting.

#### 2.2.7 Forming the SA-GelMA Support Base with GoQD

The electrospraying method was utilized with a syringe pump, an 18-gauge stainless steel nozzle, a high-voltage DC power supply, and a collector. The syringe pump was loaded with PVP and GoQD during spraying, and the needle was positioned 12.5 cm from the grounded collector. With the system optimized for a flow rate of 0.9 mL/h, the required electric field was applied at a voltage range of 20–22 kV. For the characterization process, the particles were first shot onto glass slides; their concentration was examined under an optical microscope.

#### 2.2.8 FTIR, XRD and SEM Analyses

Fourier transform infrared spectroscopy (FTIR, FT/IR-ATR 4700, USA) was employed to analyze the chemical composition of the materials used in the fabrication of the microneedle patch. Spectral analysis was performed on samples after electrospray coating with SA-GelMA, GelMA-5FU, and PVP-GoQD. Measurements were taken at room temperature within the wavelength range of 450–4000 cm^–1^ at a resolution of 4 cm^–1^.

X-ray diffraction (XRD) (Shimadzu-6100, Japan) with a Cu–Kα radiation source (*k* = 1.54060 Å) was used to characterize the crystal structures of hydrogel patches. The patterns were examined at angles ranging from 10° to 50° using a generator with a current of 40 mA and a voltage of 45 kV.

The surface morphology of both the drug-loaded and plain microneedles, the particle size of the PVP-GoQD, and the surface morphology of the dried SA-GelMA layers at different concentrations were investigated using a scanning electron microscope (SEM, EVA MA 10, Zeiss, Germany). Before imaging, all samples were coated with a thin layer of gold using a spray coater (SC7620, Quorum, UK) for 120 s. The diameter of nine microneedles and three microneedles within the needle tip radius was measured using ImageJ software to determine the needle size.

#### 2.2.9 Mechanical Tests

The mechanical properties of the microneedle patch were evaluated using a compression tester (EZ-LX, Shimadzu, Japan) in compression mode. The evaluation covered patches made of SA-GelMA/GelMA with various SA concentrations, as well as cylindrical hydrogels measuring 6 mm in height and 8 mm in diameter. These hydrogels contained 5-FU in the needle layer and PVP-GoQD in the SA-GelMA layer. A vertical compression force was applied at a constant speed of 0.1 mm/min. The force–displacement response was recorded to evaluate the compressive strength. The tests were performed in duplicate; two of the 1% SA–GelMA/GelMA and 0.5% SA–GelMA/GelMA-5FU@PVP–GoQD samples were lost during testing. The results are reported as mean ± standard deviation values.

#### 2.2.10 Swelling and Degradation Tests

The water uptake capacity of the microneedle patches was evaluated by incubating them in PBS at pH 7.4 in a thermal shaker at 37 °C. During the test, the initial weight of the patches (*W*_0_) was recorded on the first day. After a specific period (5, 15, and 30 min), the samples were removed from the excess liquid, and the wet weight (*W*_W_) of each sample was measured. The swelling ratio (*S*) was calculated using eq 2

2 $$S=\frac{Ww-W_{0}}{W_{0}}\times 100$$

in the hydrolytic degradation test, the same groups used in the swelling analysis were employed, and the initial weight (*W*_0_) was measured on the first day before treatment with PBS at pH 7.4. After treatment with PBS for the specified periods, the dry weight (*W*_d_) of the patches was calculated by removing them from PBS and drying them in a 40 °C oven for 24 h. The degradation test for all groups was performed on day 1. The degradation ratio (*D*) was calculated using eq 3

3 $$D=\frac{W_{0}-W_{d}}{W_{0}}\times 100$$

in vitro drug release evaluation.

The release profile of 5-FU from the microneedles was evaluated using a UV–vis spectrophotometer (Shimadzu UV-1280, Japan) with PBS at pH 7.4. First, a linear calibration curve was determined for 5-FU at different concentrations (0.2, 0.4, 0.6, 0.8, and 1 μg/mL) within the 190–400 nm wavelength range. 5-FU was incubated in 1 mL of PBS (pH 7.4) at 37 °C on a thermal shaker for the release analysis. The concentration of 5-FU in the hydrogel layer was determined by measuring the absorbance at 265 nm using a UV–vis spectrophotometer at predetermined time points (0.25, 0.5, 1, 12, 24, 48, 72, and 96 h). After each time point, the samples were replaced with fresh PBS for further release studies. Absorbance values were used to calculate the cumulative amount of drug released over time. Tests were performed in triplicate, and results were reported as mean ± standard deviation values.

#### 2.2.11 In Vitro Drug Release Kinetics Evaluation

The drug release kinetics of patches containing 5-FU were investigated using the five most employed mathematical models: the Korsmeyer-Peppas, zero-order, first-order, Higuchi, and Hixson–Crowell models. The Korsmeyer-Peppas (eq 4), zero-order (eq 5), first-order (eq 6), and Higuchi (eq 7) model equations are presented below

4 $$Q=Kt^{n}$$

5 $$Q=K_{0}t$$

6 $$In\left(1-Q\right)=-K_{1}t$$

7 $$Q=K_{h}t^{1/2}$$

In these mathematical equations, the kinetic constants are *K*_0_, *K*_1_, *K*, and *K*_h_. *Q* and *t* are the fractional amount of drug released at time. Additionally, *n* is the diffusion exponent, which indicates the mechanism of drug release.

#### 2.2.12 Photothermal Property Evaluation

The experimental groups (SA-GelMA/GelMA@PVP-GoQD at various concentrations) were exposed to a laser with a wavelength of 808 nm and a power density of 1 W/cm^2^ for 5 min in an Eppendorf tube containing 500 μL of PBS. PBS was used as the blank group. During the laser application, the experimental groups were recorded using a thermal camera (Fluke, Ti480 Pro, USA). Temperature measurements were taken once a min.

#### 2.2.13 Penetration Analysis of the Patch

Parafilm M was used as a skin simulant to evaluate microneedles’ penetration ability. The single-layer film, with an approximate thickness of 127 μm, was folded into eight layers to achieve a total thickness of approximately 1 mm. This multilayer film was placed on a rigid metal surface for mechanical support during testing. The dried microneedle patches were aligned with the Parafilm M surface to ensure proper contact between the microneedles and the film.

A custom device was developed to perform penetration tests by applying a controlled mechanical force to a load cell, thereby evaluating the resistance properties of various materials (Figure 2a,b). The apparatus comprises a hand-driven lead screw mechanism that translates rotational motion into precise lateral displacement of a plastic fixture. The test specimen is securely mounted on this fixture and is driven incrementally into the load cell sensor. As the specimen advances, the load cell continuously measures the applied force with high sensitivity and conveys the data via a serial communication link to a connected computer. A dedicated tare button resets the load cell before each trial to ensure accurate baseline measurements, eliminating any preloading or offset influences. During testing, the system samples force measurements at regular intervals, allowing for real-time plotting of the force–displacement curve on the computer interface. Upon completion of the test, the maximum force value recorded by the load cell corresponding to the material’s peak penetration resistance is automatically identified and logged. This functionality facilitates immediate analysis and comparison across multiple specimens. The microneedle patch was attached to the fixation part of the device and moved toward a parafilm with a thickness similar to that of pig skin. The maximum force value was then recorded as ∼45 N (Figure 2c). After placement, the microneedles were removed, and the Parafilm M layers were carefully separated. The holes generated by the microneedles in Parafilm M were imaged using an optical microscope (Olympus, USA) and analyzed using ImageJ software.

**Figure 2:**
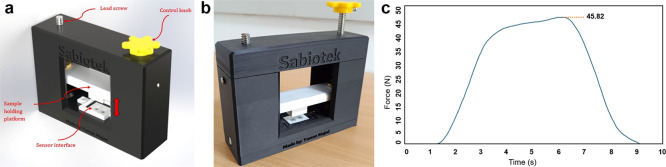
Testing device utilized for the penetration test: (a) 3D model of the device, (b) Manufactured prototype device based on the 3D model, (c) Force–time graph recorded with the accompanying software through the serial connection.

#### 2.2.14 In Vitro Cytotoxicity Analysis

The human melanoma cell line A-375 (CRL-1619, ATCC) was used. The frozen cells were thawed quickly, seeded into DMEM/F12 medium containing 10% fetal bovine serum (FBS) and 1% antibiotic (penicillin–streptomycin), and then allowed to grow in an incubator at 37 °C in a 5% CO_2_ environment. Once the cells had reached sufficient confluency, they were removed from the culture flasks using 0.25% trypsin. Afterward, 5000 cells were seeded into 96-well experimental plates. 24 h after seeding, the medium in the wells was replaced with 100 μL of medium containing the extracts from days 1 and 3 of the previously prepared and incubated (37 °C, 5% CO_2_) patches. In the control group, only the medium was replaced with fresh medium, which was also incubated under the same conditions. The experiment was terminated for cells exposed to these extracts for 24 h. The MTT (3-(4,5-dimethylthiazol-2-yl)-2,5-diphenyltetrazolium bromide) method was then applied to these cells using the protocol provided in the commercial kit (Elabscience, E-CK-A341), and cell viability rates were calculated using eq 8.

8 $$Cell\;viability\left(\%\right)=\frac{{Optical\;Density}_{570}\;Sample}{{Optical\;Density}_{570}\;Control}\times 100$$

#### 2.2.15 Statistical Analysis

Statistical analysis was performed using GraphPad Prism 9.3.0 software, as appropriate for each study. Compression analysis was performed in five replicates; swelling, degradation, and cytocompatibility were performed in at least three replicates. Data were expressed as the mean ± standard deviation (SD). The statistical analysis of compression analysis comparison data was performed using one-way ANOVA with Tukey’s multiple comparison test. The statistical analysis of the swelling, degradation, and cytocompatibility data was performed using a two-way ANOVA with a Tukey’s multiple comparison test. The height and radius of the microneedles were analyzed using an unpaired *t*-test. The graphs show appropriate symbols to indicate the p-value: ns = *p* > 0.05, * = *p* ≤ 0.05, ** = *p* ≤ 0.01, *** = *p* ≤ 0.001, **** = *p* ≤ 0.0001.

## 3 Results and Discussions

### 3.1 ^1^H NMR and TNBS

^1^H NMR spectroscopy and the TNBS assay were used to determine the degree of substitution (DS) of GelMA. ^1^H NMR spectra, analyzed using MestReNova software (v12.0.2), confirmed successful methacrylation of gelatin (Figure 3), with chemical shifts referenced to the D_2_O solvent peak at 4.79 ppm. The appearance of methacrylate vinyl proton signals at 6.1 ppm, acrylic protons at 5.4–5.7 ppm, and methyl protons at 1.9 ppm, together with the disappearance of lysine methylene signals at 3.06 and 2.90 ppm, indicated adequate substitution of lysine residues.^43,44^ DS was calculated by normalizing lysine-related signals to phenylalanine aromatic protons (7.41–7.25 ppm), yielding an estimated DS of approximately 100%. TNBS analysis showed that 90.4 ± 2.9% of primary amino groups reacted, with the slightly lower DS compared to ^1^H NMR, attributed to the limited sensitivity of NMR in detecting residual free lysine groups at high substitution levels.^39,45,46^

**Figure 3:**
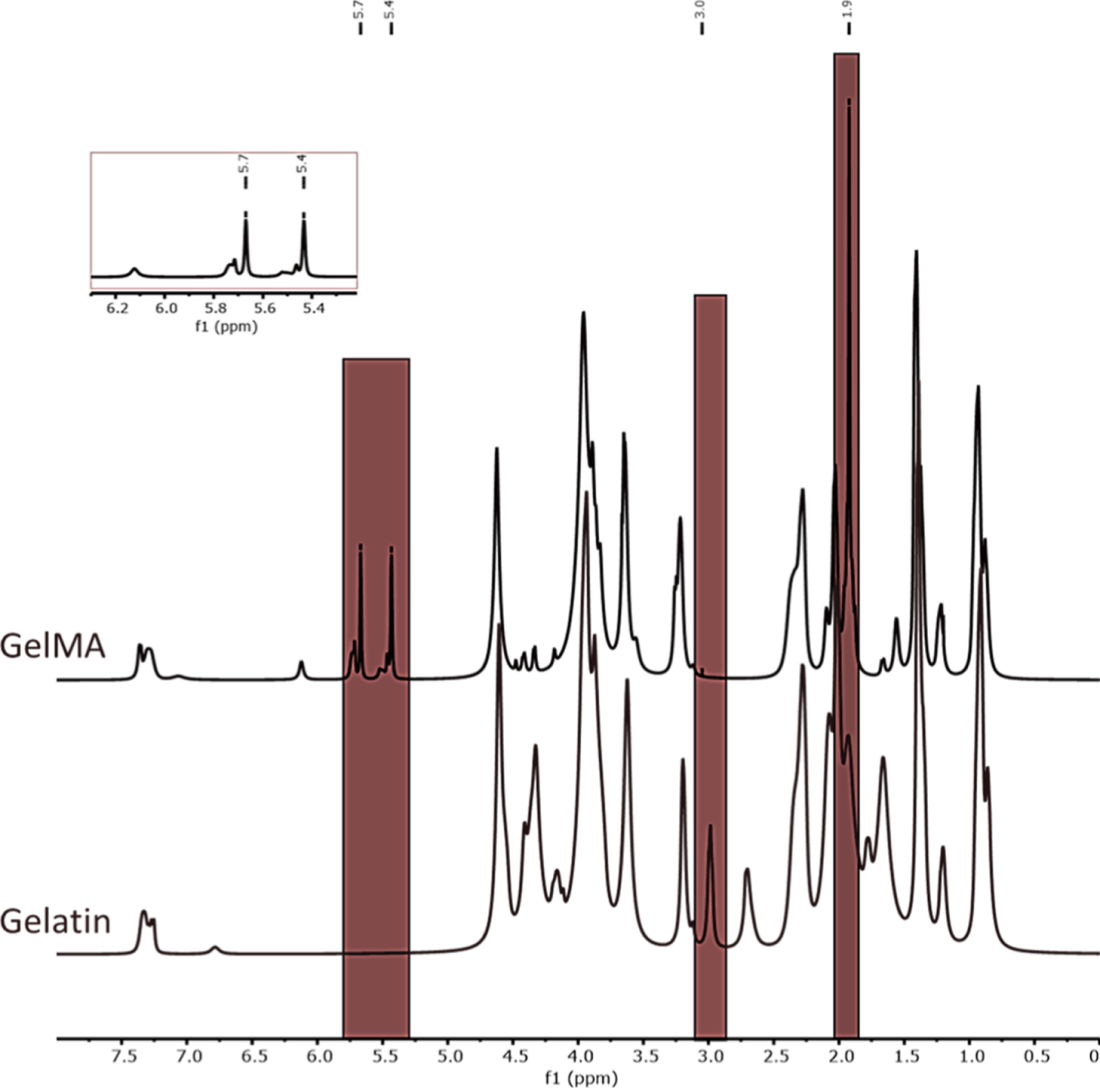
^1^H NMR Spectra of Gelatin and GelMA samples.

### 3.2 Hydrodynamic Diameter

Two distinct peaks were observed in the size distribution profile. The smaller peak at approximately 12.98 nm corresponds to individual GoQD, while the larger peak around 133.2 nm is attributed to the aggregation of GoQD.^47^ The overall Z-average particle size was 121.6 nm, with a polydispersity index (PDI) of 0.427, indicating a moderately narrow size distribution (Figure 4a).^48^ As a result, the GoQD had an acceptable particle size and PDI.

**Figure 4:**
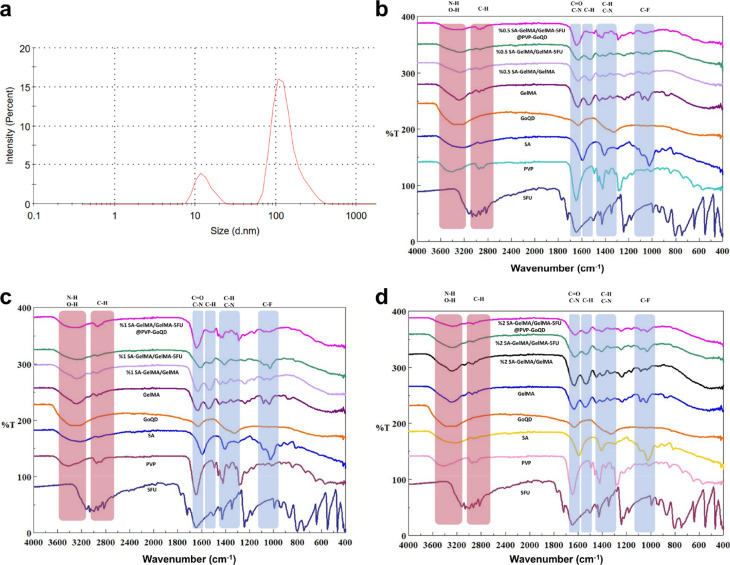
(a) Hydrodynamic size of GoQD. FTIR Spectra of the patches with (b) 0.5% SA, (c) 1% SA, (d) 2% SA.

### 3.3 Fourier Transform Infrared

FTIR analysis is essential for characterizing the chemical composition of biomaterials and revealing their suitability for use as dermal patches in biomedical applications. To this end, samples were taken from the prepared groups for each SA concentration, and their chemical structures were examined. In the 5-FU spectrum, the peak at 3121 cm^–1^ is characterized by N–H stretching vibrations; the peak at 3064 cm^–1^ by C=H stretching; the peak at 2929 cm^–1^ by CH_2_ stretching; C=O group vibrations characterize the peak at 1720 cm^–1^; the peak at 1648 cm^–1^ is characterized by C–N stretching; the peak at 1448 cm^–1^ is characterized by C–H bending; the peak at 1243 cm^–1^ is characterized by C–F stretching; and the peak at 1180 cm^–1^ is characterized by C–O vibrations.^49,50^ The FTIR spectrum of PVP can be described as follows: the peak at 3419 cm^–1^ is O–H stretching; the peak at 2951 cm^–1^ is C–H stretching; the peak at 1644 cm^–1^ is C=O stretching; the peak at 1421 cm^–1^ is C–H deformation; and the peak at 1286 cm^–1^ is C–N stretching.^37^ For SA, the peak at 3226 cm^–1^ is associated with O–H stretching vibrations, while the peak at 1593 cm^–1^ is due to COO stretching vibrations of the carboxylate groups. The peak at 1024 cm^–1^ is due to C=O stretching vibrations.^51^ The GoQD spectrum shows a broad peak at 3336 cm^–1,^ indicating O–H groups,^52^ a sharp peak at 1628 cm^–1^ corresponding to C=O stretching, and a distinct peak at 1325 cm^–1^ corresponding to C–N bending vibrations.^53^ The absence of epoxy (C–O) groups at 1221 cm^–1^ in graphene oxide confirms the presence of GoQD.^54^

For the GelMA spectrum, the prominent peak at 3294 cm^–1^ is characteristic of O–H and N–H functional groups. The peak at 1630 cm^–1^ is characteristic of N–H bending, the peak at 1537 cm^–1^ is characteristic of N–H bending groups, and the peak at 1234 cm^–1^ is characteristic of C–N stretching and N–H bending.^37,38^ First, the patches doped with 0.5% SA were evaluated. The 0.5% SA-GelMA/GelMA spectrum exhibited characteristic peaks representing O–H stretching vibrations at around 3282 cm^–1^ and a peak at 2927 cm^–1^ associated with C–H stretching. C=O stretching was observed at approximately 1628 cm^–1^ and N–H bending at 1525 cm^–1^. Additional 1448 cm^–1^ and 1238 cm^–1^ peaks can be attributed to C–H bending and C–N stretching, respectively (Figure 4b). For the 0.5% SA-GelMA/GelMA-5FU sample, slight shifts in the 1241 cm^–1^ peak were observed, which may indicate weak interactions between the SA-GelMA and 5-FU. Minor shifts in the peaks were also observed in the 0.5% SA-GelMA/GelMA-5FU@PVP-GoQD spectrum. Due to the overlapping of the characteristic bands of the chemicals used to fabricate the patch, and the very low amounts of 5-FU and PVP-GoQD, the slight shifts in the peaks in the final product, compared to SA-GelMA/GelMA, suggest the presence of 5-FU and PVP-GoQD. The same spectrum peaks were recorded in the 1% SA-doped group as in the 0.5% group. The 1% SA-GelMA/GelMA formulation exhibited distinct characteristic peaks at 3284 cm^–1^, 2935 cm^–1^, 1630 cm^–1,^ and 1531 cm^–1^, respectively. Apparent shifts were observed in the spectra of the 1% SA-GelMA/GelMA-5FU formulation and the 1% SA-GelMA/GelMA-5FU@PVP-GoQD formulation (Figure 4c). The increase in the peaks of the main functional groups with the addition of 1% SA can be attributed to the increased SA content. The same spectrum peaks were recorded in the 2% SA-doped group as in the 0.5% and 1% groups (Figure 4d).

The presence of characteristic peaks confirms the structural integrity of 5-FU, SA, and GoQD in the mixture, as well as the presence of functional groups specific to each component. FTIR analysis confirmed the presence of functional groups corresponding to SA, GELMA, 5-FU, PVP, and GoQD. This can be attributed to the minimal structural changes in SA-GELMA formulations during drug loading and composite preparation.

### 3.4 X-ray Diffraction

Due to the small size and quantity of GoQD, FTIR shifts were minimal. An XRD study was performed for further confirmation (Figure 5). The SA-GelMA/GelMA composite hydrogel exhibited typical, broad, low-intensity peaks at 2θ = 20–25° due to its amorphous structure.^55^ Subsequently, broader and higher band gaps were observed in the peaks because of 5FU@PVP-GoQD doping into SA-GelMA/GelMA. It can be speculated that the amorphous character of PVP increased the peak width.^56^ It may also have caused the increase in peak width in the GoQD, which has the same peak width.^57^ The sharp peak at 2θ = 30–35° may represent the characteristic diffraction peak of 5-FU and its crystal structure.^58^ Slight shifts in the peaks may indicate that 5FU@PVP-GoQD is integrated into the SA-GelMA/GelMA hydrogel, altering the order of the polymer chains.^59^ Therefore, it can be concluded that 5FU@PVP-GoQD was successfully incorporated into the SA-GelMA/GelMA hydrogel. Additionally, the width and height of the peaks were found to increase with increasing SA amounts in all groups. This may indicate that SA was successfully doped into GelMA.^60^

**Figure 5:**
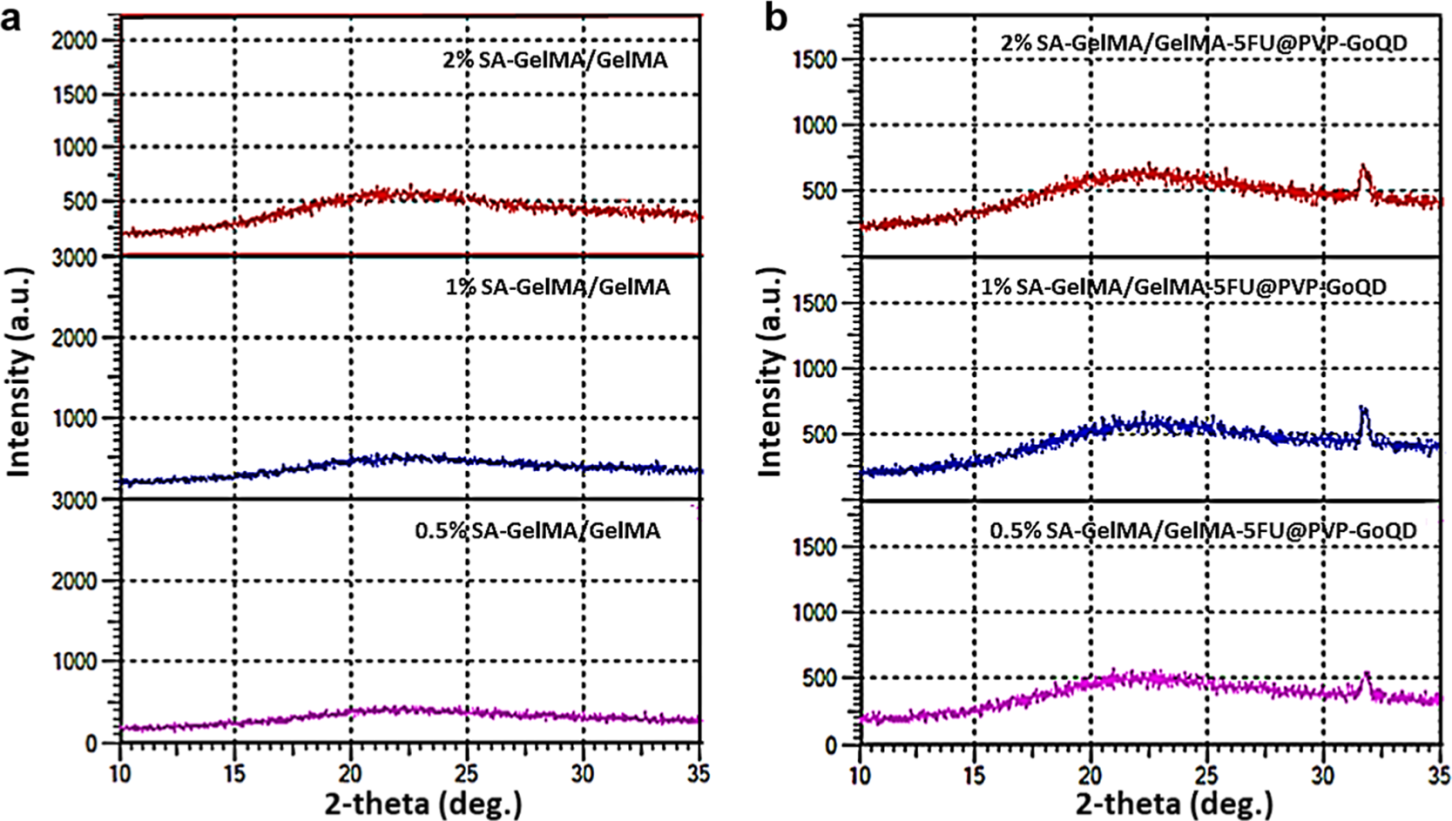
XRD patterns from (a) 0.5% SA-GelMA/GelMA, 1% SA-GelMA/GelMA, 2% SA-GelMA/GelMA; (b) 0.5% SA-GelMA/GelMA-5FU@PVP-GoQD,1% SA-GelMA/GelMA-5FU@PVP-GoQD, 2% SA-GelMA/GelMA-5FU@PVP-GoQD.

### 3.5 Morphological Analyses

The morphology of the microneedle was examined using an SEM Figure 6 shows the size and thickness of the patch. The printing process was carried out successfully within the designed dimensions.

**Figure 6:**
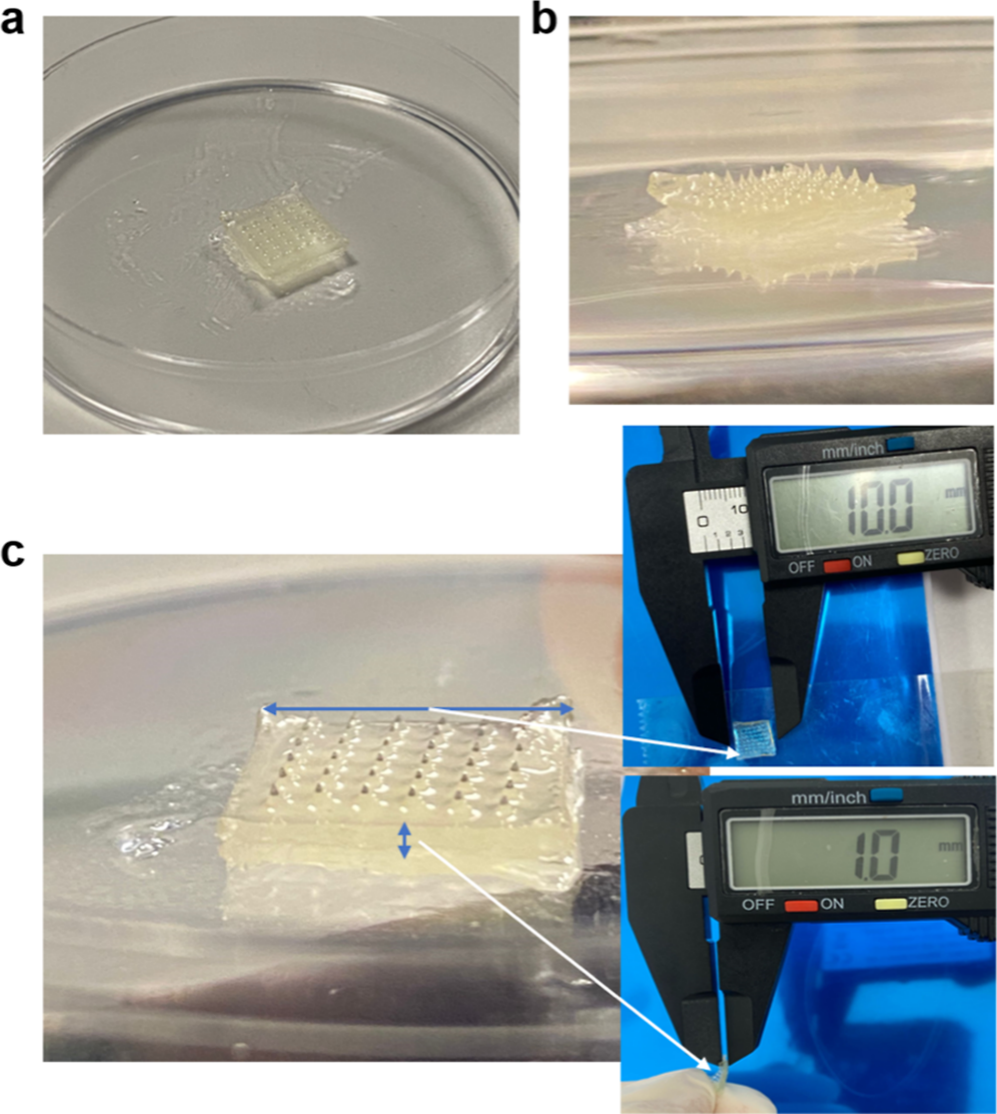
General view of the microneedle patch: (a) top view, (b) side view, (c) visualization of the patch’s thickness and size.

To prevent delamination at the 50% height point, the process was paused, and Vat 1 was removed and replaced with a second, prefilled vat (Vat 2) containing the GelMA–5FU formulation; printing was then resumed immediately to fabricate the microneedle layer directly on top of the preformed base. Using two separate vats (one per formulation) avoided in-vat draining/cleaning during the pause, minimizing material carryover. Importantly, the vat replacement time was kept very short before resuming the print, which reduced the risk of interfacial delamination at the material–switch plane by limiting surface exposure and enabling continuous layer-to-layer bonding during continuation of the same build.

The optimum concentration of PVP-GoQD was determined at different concentrations based on optical microscope images (Figure 7a). The formulation with relatively smaller and more homogeneous particle concentration (500 μL) was selected for further studies. Micrographs of the particles belonging to the PVP-GoQD coating made by electrospraying on the SA-GelMA/GelMA layer (Figure 7b). The average diameter of the particles was measured as 3.14 ± 0.846 μm (Figure 7c). The particles were distributed homogeneously on the patch to provide a stabilizing distribution of PVP.^61^ The formation of a strong bond between the polar segments of the polymer chain and the oxygen groups of the graphene sheets can stabilize GoQD, thus preventing the restacking and agglomeration of the nanosheets.^62^

**Figure 7:**
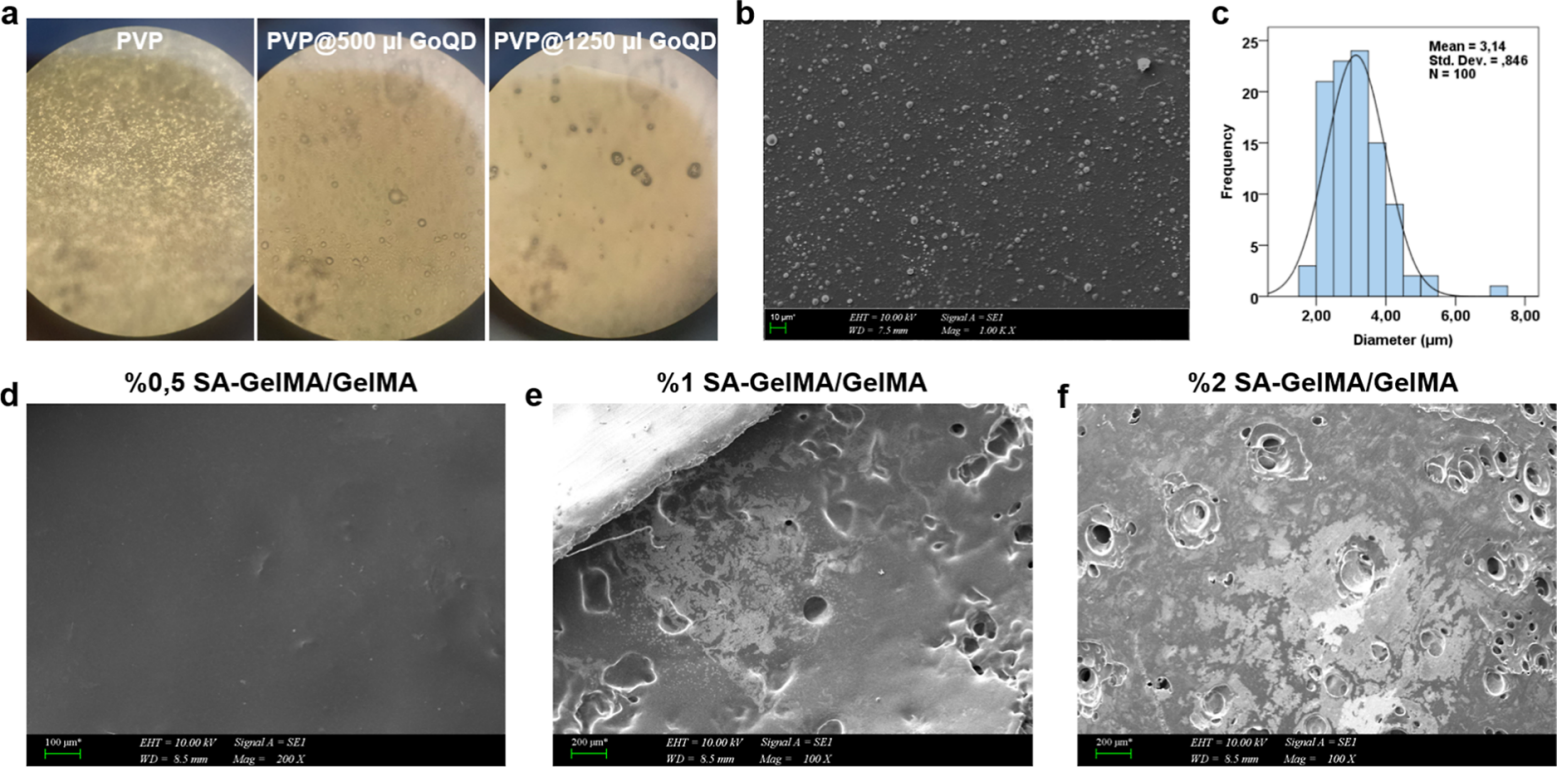
(a) Optical images of PVP and PVP-GoQD at various concentrations, (b) SEM image of 500 μL of PVP-GoQD (1.00K_X_ mag, scale bar: 10 μm), (c) Average diameter of the particles. (d–f) SEM images of SA-GelMA hydrogels with different concentrations at room temperature at the end of the third day (200_X_-100_X_ mag, scale bar: 100–200 μm).

The pore size of hydrogels is an important parameter that influences degradation profiles, mechanical properties, drug transport, cell penetration, differentiation, and high cellular activity.^63^ To examine the pore size of the SA-GelMA/GelMA layer prepared with different SA concentrations (0.5%, 1%, 2%), SEM images of the hydrogels dried at room temperature for 3 days are presented (Figure 7d–f). As the SA concentration increases, the pores become more distinct in the material’s structure. It can be inferred that the mechanical strength decreases as the pore size increases with increasing amount of SA.

The microneedles had smooth morphologies, with no air bubbles in the structure, and conical and sharp tips (Figure 8a,b). SEM images also confirmed the dimensions of the microneedles. This is important because needle height is a critical parameter that determines the microneedle penetration depth into the skin.^64^ The length of the microneedles without 5-FU was 726.7 ± 7 μm, and that of those with 5-FU was 917.6 ± 47 μm (Figure 8c). A significant difference in needle height was observed. Additionally, the microneedles containing 5-FU were close to the theoretical length of 1000 μm. Therefore, the resin prepared with 5-FU has a lower viscosity than the one without 5-FU, which provides higher permeability. This can be attributed to the shortening of the microneedles in the 5-FU-free group as the light intensity decreased below the minimum threshold value for curing during DLP printing.^65^ The height variation can be attributed to the limited UV exposure and light scattering characteristics of the reflective mirrors during photopolymerization, which may not have been sufficient to fully cure delicate structures. Additionally, the pixel intensity of the projected light may have fallen below the threshold in minor details, leading to partial curing and reduced needle height. Needle heights exceeding 600 μm are considered optimal for effective stratum corneum penetration and prevention of the “skin wrapping” effect.

**Figure 8:**
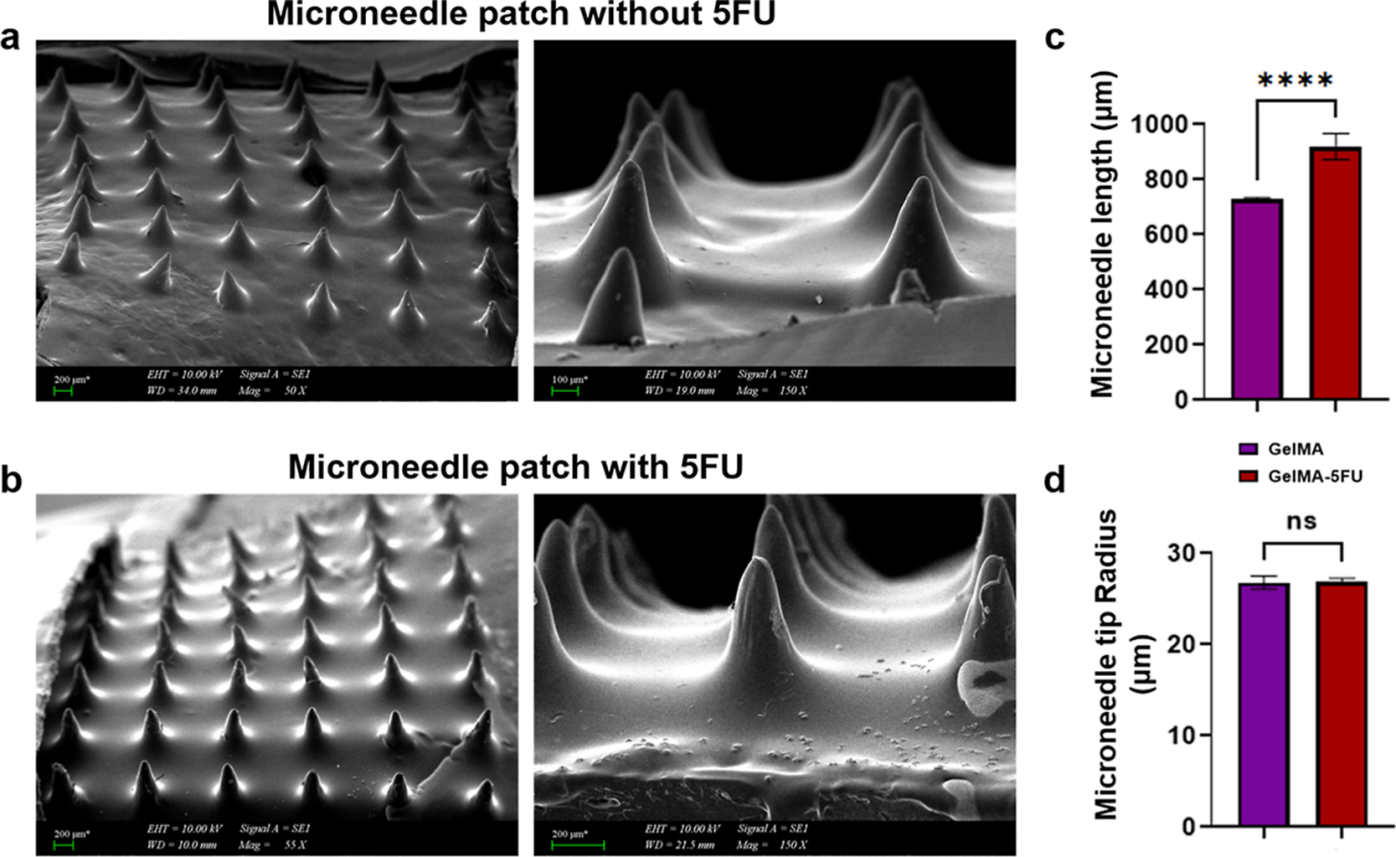
SEM images of microneedles without (a) (150_X_ mag, scale bar: 100 μm) and with (b) 5-FU (150_X_ mag, scale bar: 200 μm), (c) lengths, and (d) radii of microneedles.

The needle tip radius of the microneedles without 5-FU was 26.7 ± 0.7 μm, while that of the microneedles containing 5-FU was 26.9 ± 0.4 μm (Figure 8d). No significant difference was observed between the needle tip radii. The tip diameters obtained for both formulations (26–27 μm) fall within the 20–40 μm range reported in the literature as adequate for skin penetration.^66^

### 3.6 Mechanical Properties

The mechanical properties required for microneedle systems to penetrate the skin barrier depend on the material used, the needle geometry, and the application conditions. The most important mechanical property is that microneedles deliver the drug effectively without losing their structural integrity.^67^ The mechanical properties of the patch were evaluated using compression analysis. This analysis compared the SA-GelMA/GelMA group containing 0.5%, 1%, and 2% SA, and the SA-GelMA/GelMA-5FU@PVP-GoQD group coated with 5FU-loaded PVP-GoQD at the same ratios (Table 1 and Figure 9).

**Table 1:** Compressive Strength and Strain Values for Various Microneedle Patches

working groups of patches	compressive strength (MPa)	strain (%)	*n*
0.5% SA-GelMA/GelMA	16.57 ± 6.07	11.26 ± 5.15	5
1% SA-GelMA/GelMA	10.63 ± 1.50	12.70 ± 6.65	3
2% SA-GelMA/GelMA	5.74 ± 3.20	15.17 ± 4.60	5
0.5% SA-GelMA/GelMA-5FU@PVP-GoQD	26.73 ± 1.32	75.41 ± 33.47	3
1% SA-GelMA/GelMA-5FU@PVP-GoQD	25.52 ± 0.96	93.93 ± 13.74	5
2% SA-GelMA/GelMA-5FU@PVP-GoQD	23.92 ± 3.56	98.13 ± 18.01	5

**Figure 9:**
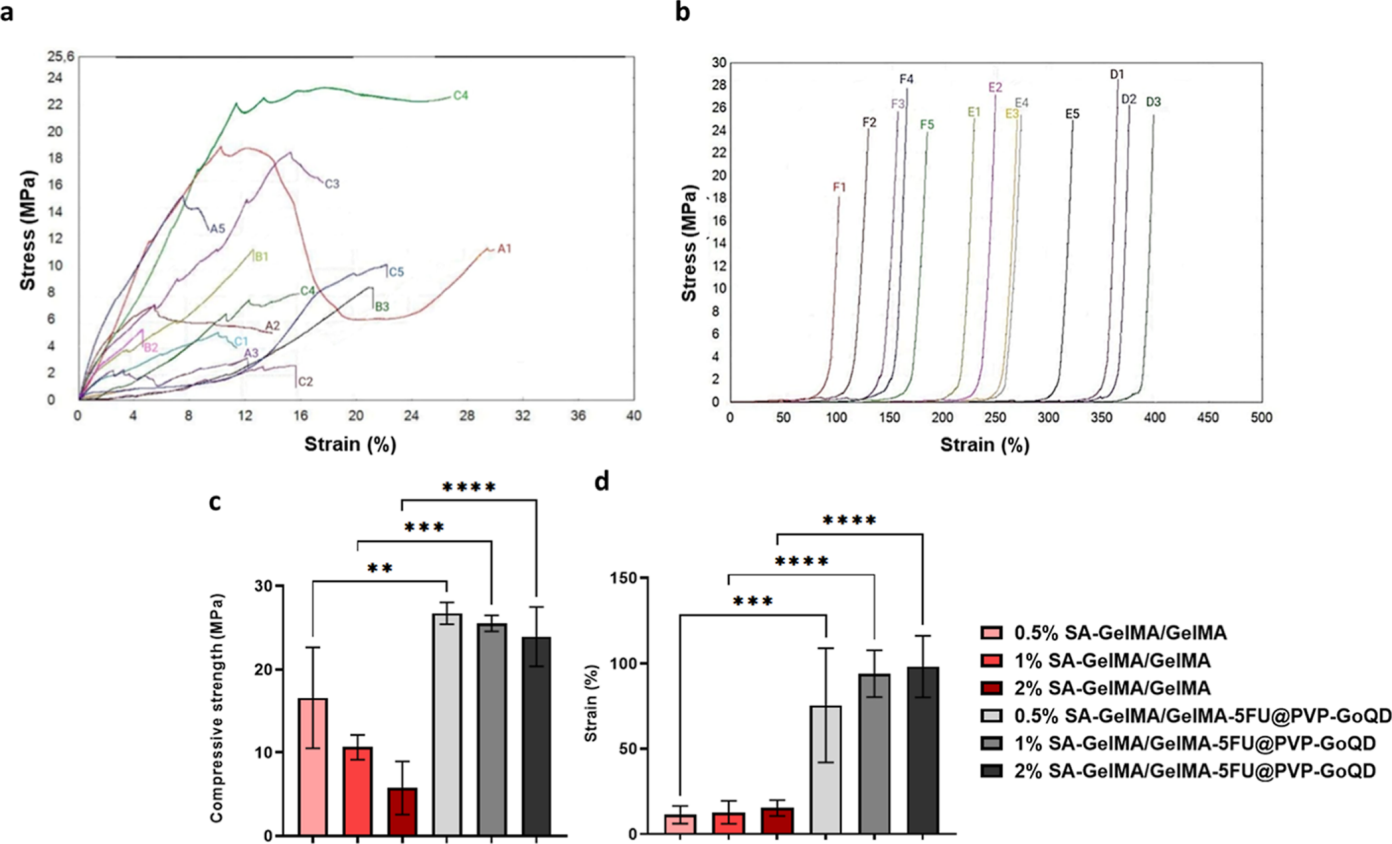
Mechanical properties of the patch: (a) stress–strain graph of the SA-GelMA/GelMA group; (A(1–5)) peaks belonging to 0.5% SA-GelMA/GelMA, (B(1–3)) peaks belonging to 1% SA-GelMA/GelMA, (C(1–5)) peaks belonging to 2% SA-GelMA/GelMA, (b) stress–strain graph of the SA-GelMA/GelMA-5FU@PVP-GoQD group; (D(1–3)) peaks belonging to 0.5% SA-GelMA/GelMA-5FU@PVP-GoQD; (E(1–5)) 1% SA-GelMA/GelMA-5FU@PVP-GoQD; (F(1–5)) 2% SA-GelMA/GelMA-5FU@PVP-GoQD, (c) compressive strength, (d) strain (%): data were analyzed using GraphPad Prism 9 software with a two-way ANOVA and a Tukey’s multiple comparison test. (** = *p* ≤ 0.01, *** = *p* ≤ 0.001, **** = *p* ≤ 0.0001; data presented as mean ± SD, *n* = 5).

All mechanical tests were initially performed with five replicates (*n* = 5) to ensure statistical reliability, as commonly reported in the literature.^68^ However, in some groups, a few samples fractured during compression, so analysis was completed with three replicates (*n* = 3). Similar sample numbers have also been reported in previous hydrogel-based mechanical studies.^69^ Therefore, the variation in sample size does not affect the validity of the results and remains consistent with standard practices in related research.

As shown in Table 1, increasing the SA content from 0.5% to 2% in the SA-GelMA/GelMA group decreased compressive strength from 16.57 ± 6.07 MPa to 5.74 ± 3.20 MPa and increased strain value from 11.26 ± 5.15% to 15.17 ± 4.60%. Upon incorporating 5-FU@PVP-GoQD nanoparticles, the compressive strength increased significantly across all SA concentrations. For instance, in the 1% SA group, compressive strength improved from 10.63 ± 1.50 MPa to 25.52 ± 0.96 MPa, and strain value increased from 12.70 ± 6.65% to 93.93 ± 13.74%. Several studies have reported mechanical properties of GelMA-based and composite hydrogel microneedles in terms of stress or modulus (MPa), especially when evaluating intrinsic material strength or normalizing force to contact area.^66,70^

Increasing the SA ratio in all groups significantly decreased the compressive strength, suggesting that SA weakens the mechanical integrity by reducing the cross-linking density in the structure. Additionally, this may be due to the GelMA hydrogel, which forms by cross-linking with covalent bonds under UV light, being unable to fully bond with the SA, as SA physically binds with ions such as Ca^2+^.^71^ Consequently, the compressive strength decreased while the strain of the patch increased. An earlier study reported that the mechanical properties of GelMA mixed with 5% alginate were higher than those of GelMA mixed with 7% alginate, where a higher alginate concentration affected the penetration of UV light for cross-linking.^69^ Conversely, the compressive strength increased significantly in the SA–GelMA/GelMA-5FU@PVP-GoQD group coated with 5-FU-loaded PVP-GoQD particles, which can be interpreted as strong interactions between SA–GelMA/GelMA functional groups resulting from coating with 5-FU and PVP-GoQD particles. This can be attributed to the fact that optimum amounts of GoQD improve the mechanical properties of GelMA hydrogels.^72,73^ Incorporating carbon nanomaterials such as GoQD into hydrogels can significantly enhance their mechanical, electrical, and thermal properties.^74^ Additionally, it was observed that the decrease in compressive strength caused by increasing the amount of SA continued, even after coating with 5-FU-loaded PVP-GoQD. Examining the strain data revealed a significant increase in ductility across all groups, accompanied by an increase in the SA ratio. The strain values in the SA–GelMA/GelMA-5FU@PVP-GoQD samples were significantly higher than those without 5FU@PVP-GoQD, which can be attributed to the fact that 5FU@PVP-GoQD acts as a cross-linker in GelMA by binding to GelMA and increasing its mechanical strength.^75^ The mechanical analysis revealed that the patches were sufficiently strong to penetrate the skin barrier without deforming. The high strain values of the SA–GelMA/GelMA-5FU@PVP-GoQD samples indicate that the hydrogel is flexible, which can provide a skin-compatible and comfortable application.^76^ Although higher SA content slightly accelerated degradation due to weaker network stability, the coated and drug-loaded microneedles exhibited slower mass loss and maintained their shape under physiological conditions. Furthermore, penetration tests confirmed that all microneedle groups, despite differences in mechanical strengths, successfully pierced multiple Parafilm layers without observable deformation or breakage. These results suggest that the optimized SA–GelMA/GelMA-5FU@PVP-GoQD formulation offers stability during skin application by striking an effective balance between mechanical strength and controlled degradation.

### 3.7 Swelling and Degradation Tests

The swelling behavior of microneedles is a critical factor in determining their effectiveness, as this property directly affects both drug release and tissue interaction.^77^ 0.5%, 1%, and 2% SA–GelMA/GelMA patches were incubated in PBS for 5, 15, and 30 min, after which their mass changes were analyzed. The fastest increase in swelling rates occurred within the first 5 min, after which the swelling capacity saturated at 15 min. The highest swelling percentages were observed in the 0.5% SA–GelMA/GelMA (∼285%), 1% SA–GelMA/GelMA (∼280%), and 2% SA–GelMA/GelMA (∼253%) groups. No statistically significant decrease was observed among the groups, while it can be inferred that swelling percentages decreased as the amount of SA increased. The swelling percentages of the 0.5%, 1%, and 2% SA–GelMA/GelMA-5FU@PVP-GoQD patches were higher than those of the groups without 5FU@PVP-GoQD. Additionally, the swelling percentages decreased as the SA amount increased. The swelling percentages were 337% for 0.5% SA–GelMA/GelMA-5FU@PVP-GoQD, 313% for 1% SA–GelMA/GelMA-5FU@PVP-GoQD, and 303% for 2% SA–GelMA/GelMA-5FU@PVP-GoQD (Figure 10a). GelMA-based MNs can absorb interstitial fluid in the skin due to their porous, hydrophilic structure, providing an opportunity for substance exchange between the microneedles and the skin.^27^ Therefore, it can be concluded that the microneedle structures prepared within the scope of the study are suitable for absorbing interstitial fluid from the skin. This provides an ideal environment for the release of 5-FU from the needle tip and PVP-GoQD from the base.

**Figure 10:**
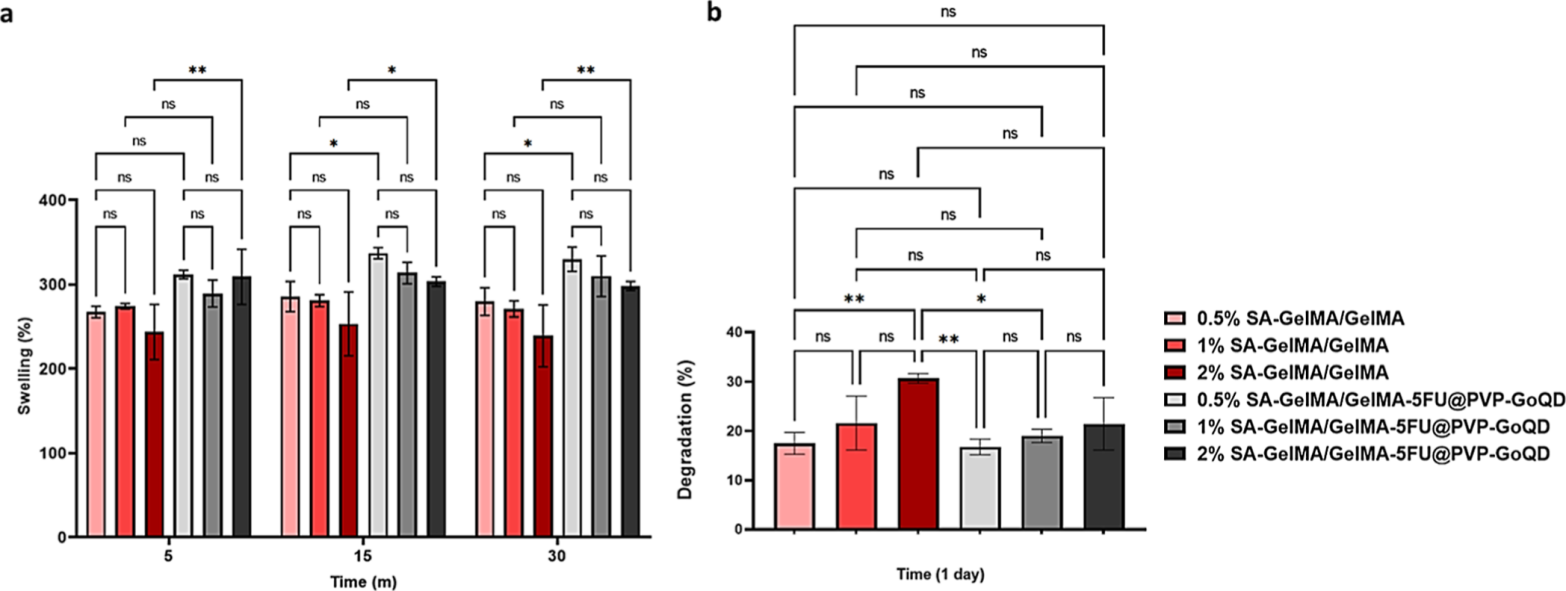
a) The swelling behavior of the patch after incubation in PBS for different time intervals. The data were analyzed using GraphPad Prism 9 software with a two-way ANOVA and a Tukey’s multiple comparison test. (ns = *p* > 0.05; * = *p* ≤ 0.05; ** = *p* ≤ 0.01; data presented as mean ± SD, *n* = 3). (b) The degradation behavior of the patch after incubation in PBS for different time intervals. The data were analyzed using GraphPad Prism 9 software with a two-way ANOVA and a Tukey’s multiple comparison test. (ns = *p* > 0.05; * = *p* ≤ 0.05; ** = *p* ≤ 0.01; *** = *p* ≤ 0.001; **** = *p* ≤ 0.0001; data presented as mean ± SD, *n* = 3).

Studies have shown that hydrogel-based microneedles swell rapidly within the first few minutes of being immersed in PBS. For instance, GelMA/PVA microneedles swell considerably within 5–10 min in PBS, which highlights the importance of measuring hydration levels early on for microneedle function.^78^ Consistent with our findings, Luo et al. reported that GelMA-based microneedles exhibit rapid swelling behavior shortly after exposure to aqueous environments, and that early stage hydration is sufficient to facilitate interstitial fluid uptake and drug diffusion without requiring long-term equilibrium swelling.^40^

Although SA is inherently hydrophilic, the observed reduction in swelling with increasing SA content in the microneedle patch may arise from multiple factors. First, higher SA levels can alter the overall polymer composition, leading to a more compact network structure with reduced free volume available for water uptake, which may interfere with GelMA’s UV-mediated covalent cross-linking and result in a denser, less swellable hydrogel network. Second, high SA content may promote increased ionic interactions and chain entanglement, which can restrict polymer chain mobility and limit swelling despite SA’s hydrophilicity. Previous studies on SA-based composite hydrogels have reported that increasing the alginate content beyond optimal levels can decrease the hydrogel’s water absorption capacity, likely due to a more constrained polymer network and reduced mesh size available for fluid infiltration.^69,79^

The hydrolytic degradation behavior of the patches with different formulations is provided in Figure 10b. The degradation behaviors of the 0.5%, 1%, and 2% SA-GelMA/GelMA patches were investigated after incubation in a PBS solution at 37 °C for 1 day. The lowest and highest degradation percentages were observed at the end of the first day: 0.5% SA–GelMA/GelMA (∼16%), 1% SA-GelMA/GelMA (∼22%), and 2% SA–GelMA/GelMA (∼31%), respectively. As the amount of SA increased, the degradation percentages also increased. The degradation percentages of the 0.5%, 1%, and 2% SA–GelMA/GelMA-5FU@PVP-GoQD patches were higher than those of the patches without 5FU@PVP-GoQD. Additionally, as the amount of SA increased, the degradation percentages also increased. It can therefore be concluded that the patches are suitable for long-term use. Previous studies on GelMA-based microneedles and diagnostic microneedle systems have primarily focused on early stage structural integrity and functional degradation, rather than long-term bulk degradation. In line with these reports, degradation analysis in the present study was limited to the time frame relevant to microneedle application.^40,78^ The reduction in swelling observed with increasing SA content is consistent with previous findings in composite GelMA-alginate systems, where polymer concentration and network interactions modulate both swelling and degradation behavior.

### 3.8 In Vitro Release of 5-Fluorouracil

In vitro drug release studies were conducted to evaluate the release behavior of 5-FU from GelMA–5FU hydrogels under physiological conditions. The experiments were performed in PBS (pH 7.4) at 37 °C for 96 h, and the amount of released 5-FU was quantified using UV–Vis spectroscopy at 265 nm. A linear calibration curve was established in the concentration range of 0.2–1 μg/mL, demonstrating excellent linearity (*R* ^2^ = 0.9985) (Figure 11a,b).

**Figure 11:**
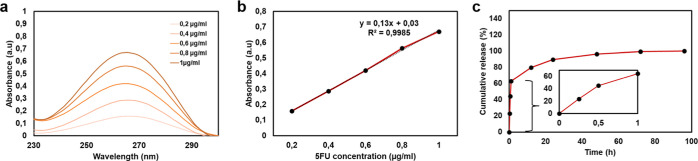
In vitro drug release profile of GelMA-5FU patches. (a) Spectra of 5-FU at different concentrations, (b) calibration curve of 5-FU, (c) time-dependent release of 5-FU from the patches.

GelMA–5FU hydrogels exhibited a distinct biphasic release profile (Figure 11c). An initial burst release of 63% ± 0.665% occurred within the first hour, followed by a sustained release phase that continued until complete drug release was achieved at 96 h. The pronounced burst release is attributed to the rapid diffusion of weakly bound or surface-associated 5-FU, whereas the subsequent slower release phase is governed by stronger drug–hydrogel interactions and matrix-controlled diffusion mechanisms.^80^

Despite the initial rapid release, incorporation of 5-FU into the GelMA-based microneedle system enabled prolonged drug release over 96 h, underscoring its potential for localized and sustained drug delivery applications.

### 3.9 Release Kinetics of 5-Fluorouracil

The release kinetics of 5-FU from GelMA-5FU patches were analyzed to elucidate the underlying mechanisms of drug release. Controlled release of bioactive agents can enhance their effectiveness and facilitate clinical applications. Drug release is the process by which drug molecules transfer from their initial location within a polymeric system to the outer surface and subsequently into the release medium. Multiple factors, including the physicochemical properties of the drug, structural characteristics of the polymer, the release medium, and interactions among these elements, influence this process.^81^

To quantitatively assess the release mechanism, the experimental data were fitted to commonly used kinetic models, including zero-order, first-order, Higuchi, and Korsmeyer–Peppas models (Figure 12). The corresponding kinetic constants and regression coefficients (*R* ^2^) are summarized in Table 2. Among the evaluated models, the Higuchi model provided the best fit, exhibiting the highest correlation coefficient (*R* ^2^ = 0.9765). The Higuchi plot showed a linear relationship described by the equation *y* = 63.649_x_ – 2.6165, indicating that the release of 5-FU from GelMA during the sustained phase is predominantly governed by diffusion-controlled transport. In this model, dissolved drug molecules diffuse through the hydrated polymeric network, while undissolved drug molecules gradually dissolve and diffuse into the surrounding medium over time.^82,83^

**Figure 12:**
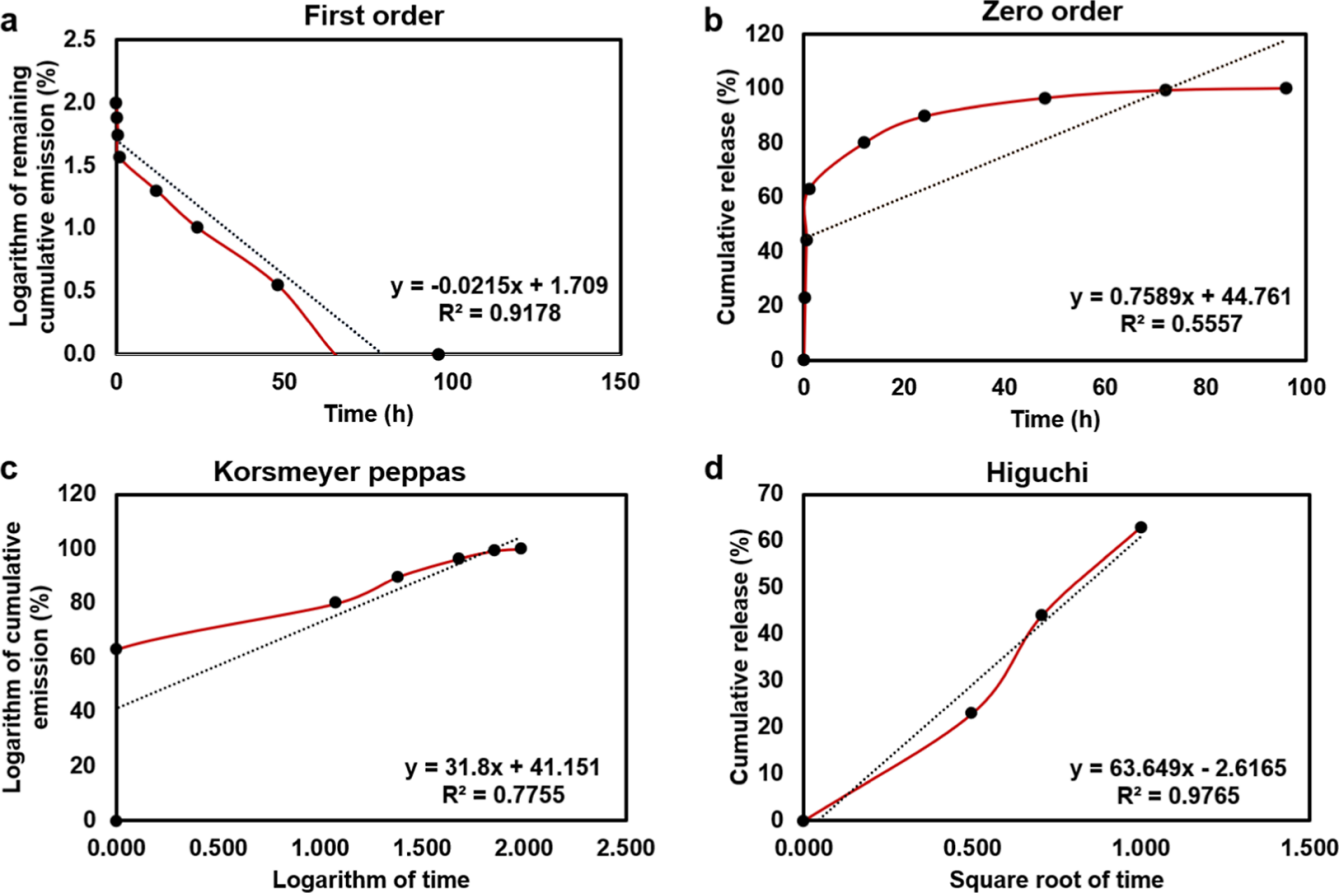
Release kinetics of different mathematical models for GelMA-5FU: (a) first-order model, (b) zero-order model, (c) Korsmeyer–Peppas model, (d) Higuchi model.

**Table 2:** Results of Mathematical Drug Release Modeling for GelMA-5FU Patches

	korsmeyer–-peppas		zero-order		first-order		higuchi	
	*R* ^2^	*n*	*R* ^2^	*K*_0_	*R* ^2^	*K*_1_	*R* ^2^	*K*_h_
GelMA-5FU	0.7755	31.8	0.5557	0.7589	0.9178	–0.0215	0.9765	63.65

Additional mechanistic insight was obtained from the Korsmeyer–Peppas model. The release exponent (*n*) for GelMA–5FU was found to be greater than 1, suggesting a Super Case II transport mechanism. This implies that drug release is influenced not only by diffusion but also by polymer chain relaxation, swelling, and structural reorganization of the hydrogel network. The hydrophilic nature of GelMA likely facilitates swelling-induced polymer relaxation, contributing to the observed release behavior.^84^

Overall, the kinetic analysis demonstrates that although an initial burst release occurs, the subsequent release of 5-FU from GelMA–5FU patches is effectively modulated by diffusion-controlled transport in combination with polymer relaxation mechanisms. This synergistic behavior results in prolonged drug release over a period of up to 96 h.

Theoretical drug loading was 20 mg/mL (≈9.1 wt % relative to total solids); however, encapsulation efficiency was not calculated, as the drug was incorporated directly into the hydrogel precursor without a separable carrier phase. In the current fabrication strategy, 5-FU was directly dissolved in the GelMA precursor solution before DLP printing, resulting in the bulk incorporation of the drug within the hydrogel matrix rather than encapsulation within a distinct carrier system, such as nanoparticles or microparticles. Consequently, the conventional concept of “encapsulation efficiency”, which is typically determined using supernatant-based quantification methods, is not directly applicable and was therefore not assessed.

Instead, the theoretical drug loading was defined based on the formulation used during printing, corresponding to 20 mg of 5-FU per mL of GelMA precursor (approximately 9.1 wt % relative to the total solid content). Effective drug incorporation was further supported by the in vitro release profile, which demonstrated complete drug release within 96 h, as well as by the observed cytotoxic activity against A375 melanoma cells.

Additionally, in vitro drug release and release kinetics were systematically investigated, and the results were found to be consistent with previously reported data for similar hydrogel-based drug delivery systems.

### 3.10 In Vitro Penetration Analysis

The penetration properties of the microneedles were evaluated using an 8-layer Parafilm model, where each layer had a thickness of 127 μm.^85^ By manually applying thumb force, the perforated layers were analyzed using optical microscopy (Figure 13a). In the case of microneedles without 5-FU, all the needles perforated the first layer. 65.31 ± 1.96% of the microneedles perforated the second layer, whereas the value was 48.98 ± 2.95% for the third layer. The third layer corresponds to a penetration depth of approximately 381 μm, indicating that approximately 51.65% of the average needle height (∼726 μm) was inserted. In contrast, microneedles containing 5-FU exhibited a deeper penetration profile. All needles perforated the first and second layers. In the third layer, 85.71% ± 2.24% of the microneedles formed holes, while in the fourth layer, 51.02% ± 2.63% formed holes (Figure 13b). This indicates a penetration depth of approximately 500 μm, meaning that approximately 54.52% of the average needle height (∼917 μm) was inserted. Thus, it can be concluded that the microneedles can easily penetrate the dermis and deliver 5-FU without causing pain. In Parafilm tests, three layers or fewer may be suitable for epidermal applications, while at least four layers may be suitable for targeting the dermis.^86^ This corresponds to a penetration depth of around 500 μm. The improved penetration profile of drug-loaded microneedles can be associated with increased needle height and possibly increased mechanical strength. The results are consistent with those of the SEM and mechanical analyses.

**Figure 13:**
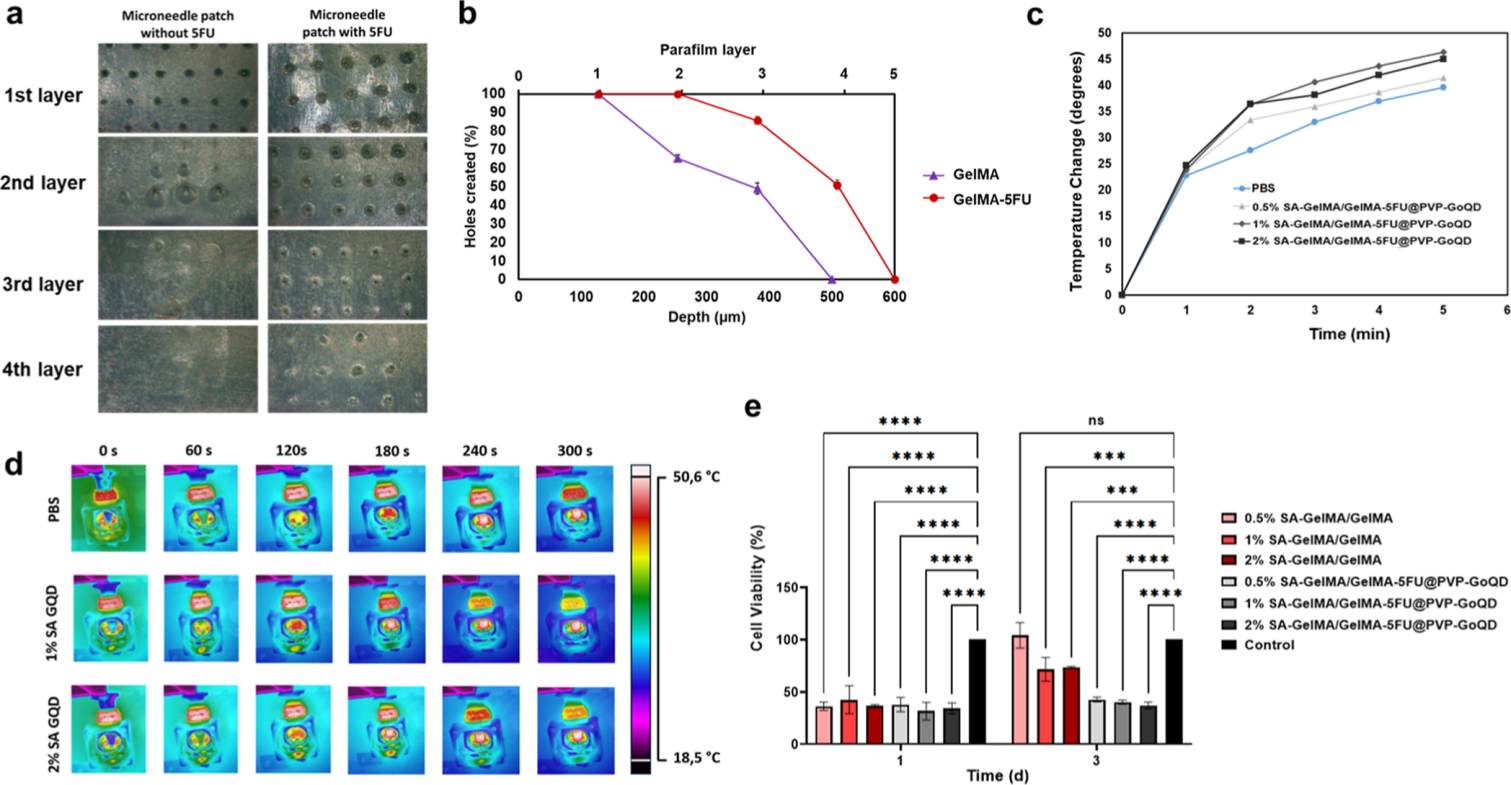
a) Optical image showing the penetration of patches with and without 5-FU, (b) depth graphs resulting from penetration. (c) photothermal heating curve, (d) thermal camera images of SA–GelMA/GelMA-5FU@GoQD hydrogels under 808 nm laser (1 W/cm^2^) irradiation, (e) MTT results for A375 cells on days one and three. The data were analyzed using GraphPad Prism 9 software with a two-way ANOVA and a Tukey’s multiple comparison test. (ns = *p* > 0.05; *** = *p* ≤ 0.001; **** = *p* ≤ 0.0001; data presented as mean ± SD, *n* = 3).

### 3.11 Photothermal Properties

Patches functionalized with PVP-GoQD enabled photothermal heating upon exposure to NIR light (808 nm, 1 W/cm^2^). The temperature increase over time was monitored using a thermal camera, revealing that the GoQD patches reached 46.3 °C within 5 min (Figure 13c). Temperatures of 40–47 °C, produced during photothermal therapy, can permanently damage proteins in cancer cells and disrupt DNA function, leading to the apoptosis of these cells. Additionally, temperatures above 50 °C can induce necrosis in cancer cells, resulting in rapid cell death in both cancerous and healthy tissue cells.^87^ GoQD can absorb laser energy and convert it into localized heat.^88,89^ Therefore, it can be concluded that the patches containing PVP-GoQD have photothermal properties, and that the first 5 min are the optimum time for NIR treatment. Real-time images recorded by the thermal camera also confirmed the photothermal efficiency of the hydrogels (Figure 13d).

### 3.12 Cytotoxicity

Cell viability was assessed by the MTT assay following exposure of A375 cells to patch extracts after 1 and 3 days. All data were normalized to untreated control cells. Following 24 h incubation with the 3 day extracts, the average cell viability remained above 70% for all groups, which were 104 ± 12.2% for 0.5% SA-GelMA/GelMA, 72 ± 11.36% for 1% SA-GelMA/GelMA, and 74 ± 0.7% for 2% SA–GelMA/GelMA, indicating noncytotoxicity in accordance with ISO 10993-5. In contrast, patches containing 5FU@PVP-GoQD resulted in a pronounced decrease in cell viability, yielding values of 43 ± 2.31% for 0.5% SA–GelMA/GelMA-5FU@PVP-GoQD, 40 ± 1.97% for 1% SA–GelMA/GelMA-5FU@PVP-GoQD, and 37 ± 3.17% for 2% SA–GelMA/GelMA-5FU@PVP-GoQD (37 ± 3.17) (Figure 13e). These values fell well below the ISO-defined cytotoxicity limit and were significantly lower than those of the control group (*p* < 0.0001, day 3). The observed cytotoxicity is consistent with the established anticancer activity of 5-FU, a clinically used chemotherapeutic agent for skin cancer treatment^90^, and is corroborated by the response of A375 melanoma cells.

## 4 Conclusion

In this study, we developed a minimally invasive 3D DLP–printed hydrogel microneedle patch that combines controlled 5-fluorouracil (5-FU) delivery with NIR-responsive photothermal functionality to enable synergistic chemo-photothermal therapy for melanoma. The bilayer design, consisting of a SA–GelMA support layer and 5-FU–loaded GelMA microneedles, provided suitable mechanical integrity and geometry for transdermal application. The microneedles (917.6 ± 47 μm in height, 26.9 ± 0.4 μm tip radius) achieved effective insertion into skin simulants, supporting their ability to overcome the skin barrier for localized drug delivery. In vitro release studies demonstrated sustained 5-FU delivery over 96 h, while GoQD-based photothermal activation generated rapid hyperthermia under 808 nm irradiation, reaching 46.3 °C within 5 min. Importantly, the drug-loaded and photothermal-functionalized patches significantly reduced A375 melanoma cell viability, highlighting their potential as a localized therapeutic strategy. Future studies incorporating additional mechanistic in vitro assays and in vivo melanoma models will be essential to validate therapeutic efficacy, safety, and translational feasibility.

## Supplementary Material



## Data Availability

The data sets generated during and/or analyzed during the current study are available from the corresponding author on reasonable request.
