# Supplementary material for: 3D-Printed Microneedle Patch for the Treatment of Melanoma via Synergistic Chemotherapy and Photothermal Therapy
Source: ACS Appl Bio Mater. 2026 Jan 26;9(4):1888–905. doi: 10.1021/acsabm.5c01606 (PMC12914633; doi:10.1021/acsabm.5c01606)
Supplement: Supplementary file 1 [file mt5c01606_si_001.pdf]

# *Supporting Information*

# *for*

## 3D-Printed Microneedle Patch for the Treatment of Melanoma via Synergistic Chemotherapy and Photothermal Therapy

Hilal Yilmaz[1,2,3,*], Louna Karzoun[1,2,4], Berfin Ilayda Ozturk Guzelcan[5,6], Hakan Sahin[2,7], Yagmur Kazancioglu[1], Mohammad Yaman Habra[2,8], Esra Yuca Yilmaz[2,4], Elif Guzel[2,7], Oguzhan Gunduz[2,3,9], Yavuz Nuri Ertas[5,6,*], Cem Bulent Ustundag[1,2,3,*]

[1] Department of Bioengineering, Faculty of Chemical and Metallurgical Engineering, Yildiz Technical University, Istanbul 34210, Türkiye

[2] Health Biotechnology Joint Research and Application Center of Excellence (SABIOTEK), Esenler, Istanbul 34220, Türkiye

[3] Center for Nanotechnology & Biomaterials Application and Research (NBUAM), Marmara University, Istanbul 34722, Türkiye

[4] Department of Molecular Biology and Genetics, Faculty of Science and Literature Department, Yildiz Technical University, Istanbul 34210, Türkiye

[5] Department of Biomedical Engineering, Erciyes University, Kayseri 38039, Türkiye

[6] ERNAM–Nanotechnology Research and Application Center, Erciyes University, Kayseri 38039, Türkiye

[7] Department of Histology and Embryology, Cerrahpasa Faculty of Medicine, Istanbul University-Cerrahpasa, Istanbul 34098, Türkiye

[8] Department of Biomedical Engineering, Faculty of Electrical and Electronics, Yildiz Technical University, Istanbul 34220, Türkiye

[9] Department of Metallurgical and Materials Engineering, Faculty of Technology, Marmara University, Istanbul 34722, Türkiye

*Corresponding Authors:

Hilal Yilmaz, hilaltoptas44@gmail.com

Yavuz Nuri Ertas, yavuznuri@gmail.com

Cem Bulent Ustundag, cbustundag@gmail.com

**XRD**

XRD patterns confirmed the presence of all individual raw materials used in the fabrication of the microneedle patch. GelMA exhibited a broad diffraction halo centered at approximately $2\theta \approx 20°$, which is characteristic of gelatin-based polymers with a predominantly amorphous structure [1]. SA showed characteristic diffraction features with broad peaks around $2\theta \approx 13$–14° and 21–22°, consistent with previously reported XRD patterns of alginate-based materials [2]. PVP displayed its typical amorphous diffraction pattern, with broad features observed at approximately $2\theta \approx 13°$ and 21°, confirming its non-crystalline nature [3]. GO exhibited a distinct diffraction peak at approximately $2\theta \approx 10$–11°, corresponding to the (001) plane of oxidized graphene layers, while the low-intensity background at higher angles reflects structural disorder and the presence of residual graphitic domains within the GO sheets [4]. GoQD showed a broad diffraction band centered around $2\theta \approx 24$–26°, which is commonly attributed to their small lateral size and disordered graphitic structure [5]. In contrast, 5-FU exhibited sharp and intense diffraction peaks in the range of $2\theta \approx 28$–31°, confirming its crystalline structure and good agreement with reported reference patterns [6]. Overall, these characteristic diffraction features validate the successful identification of GelMA, SA, PVP, GO, GoQD, and 5-FU as the raw materials employed in the microneedle system.

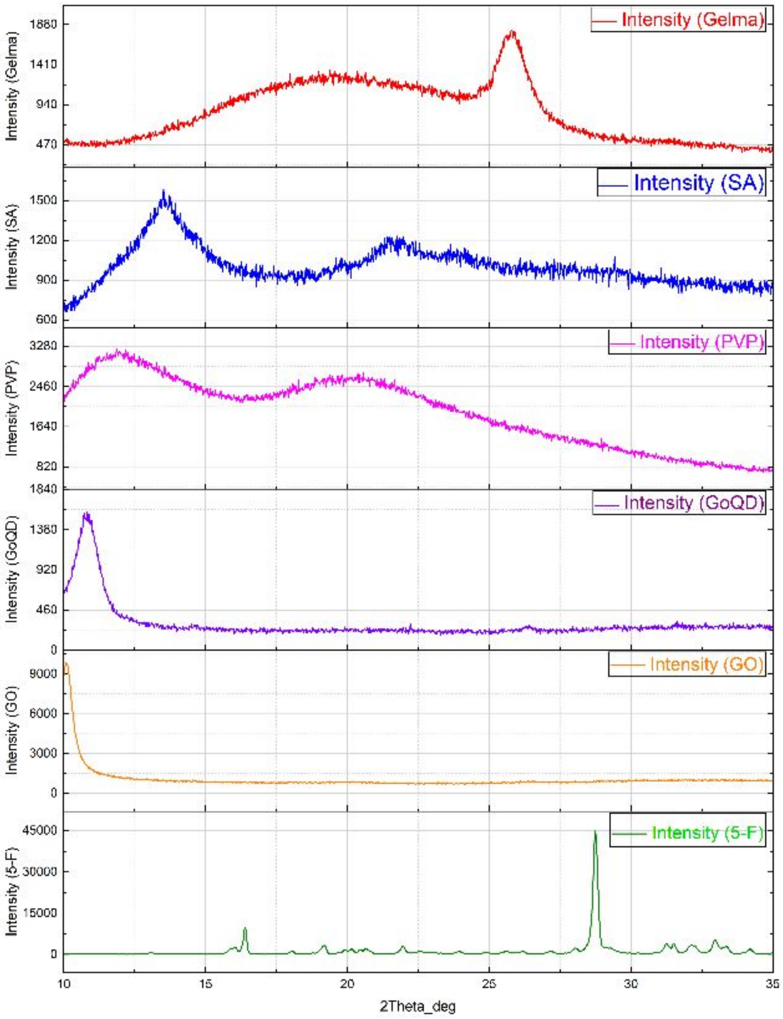


**Figure S1.** XRD patterns of raw materials

## References


[1] Van Den Bulcke, A. I.; Bogdanov, B.; De Rooze, N.; Schacht, E. H.; Cornelissen, M.; Berghmans, H. Structural and rheological properties of methacrylamide-modified gelatin hydrogels. *Biomacromolecules* **2000**, *1* (1), 31–38.

[2] Pawar, S. N.; Edgar, K. J. Alginate derivatization: A review of chemistry, properties, and applications. *Biomaterials* **2012**, *33* (11), 3279–3305.

[3] Teodorescu, M.; Bercea, M. Poly(vinylpyrrolidone)–A versatile polymer for biomedical and beyond medical applications. *Polym.-Plast. Technol. Eng.* **2015**, *54* (9), 923–943.

[4] Dreyer, D. R.; Park, S.; Bielawski, C. W.; Ruoff, R. S. The chemistry of graphene oxide. *Chem. Soc. Rev.* **2010**, *39* (1), 228–240.

[5] Sun, H.; Wu, L.; Wei, W.; Qu, X. Recent advances in graphene quantum dots for sensing. *Mater. Today* **2013**, *16* (11), 433–442.

[6] Mureșan-Pop, M.; Cherecheș, G.; Borodi, G.; Fischer-Fodor, E.; Simon, S. Structural characterization of 5-fluorouracil–piperazine new solid forms and evaluation of their antitumor activity. *J. Mol. Struct.* **2020**, *1207*, 127842.
